# Mass spectrometry imaging reveals spatial metabolic variation and the crucial role of uridine metabolism in liver injury caused by *Schistosoma japonicum*

**DOI:** 10.1371/journal.pntd.0012854

**Published:** 2025-02-11

**Authors:** Qingkai Xue, Xiangyu Zhou, Yuyan Wang, Yiyun Liu, Xiaojing Li, Chunrong Xiong, Xinjian Liu, Yuzheng Huang

**Affiliations:** 1 National Health Commission Key Laboratory of Parasitic Disease Control and Prevention, Jiangsu Provincial Key Laboratory on Parasite and Vector Control Technology, Jiangsu Institute of Parasitic Diseases, Wuxi, Jiangsu, China; 2 Experimental Center of Clinical Research, The First Affiliated Hospital of Anhui University of Chinese Medicine, Hefei, Anhui, China; 3 Tropical Diseases Research Center, Nanjing Medical University, Wuxi, Jiangsu, China; 4 School of Public Health, Nanjing Medical University, Nanjing, Jiangsu, China; 5 Department of Pathogen Biology, Key Laboratory of Antibody Techniques of National Health Commission, Nanjing Medical University, Nanjing, Jiangsu, China; Texas Biomedical Research Institute, UNITED STATES OF AMERICA

## Abstract

Schistosomiasis is the second most important parasitic disease worldwide. *Schistosomiasis japonica* is a unique species endemic to southern China, and schistosomiasis is characterized by severe liver injury, inflammation, liver granuloma, and subsequent liver fibrosis. However, the pathological mechanism of this disease remains unclear. Mass spectrometry imaging (MSI) is a versatile technique that integrates the molecular specificity of mass spectrometry (MS) with spatial imaging information, which could provide an accurate method for observing disease progression. In this study, we used an air flow-assisted desorption electrospray ionization (AFADESI-MSI) platform to detect a wide range of metabolites and visualize their distribution in the liver tissue of mice infected with S*chistosoma japonicum*. In the negative ion mode analysis, 21 and 25 different metabolites were detected in the early and chronic stages of infection, respectively. Thirteen characteristic metabolites and 3 metabolic pathways related to disease development may be involved in the chronicity of schistosomiasis. There were more than 32 and 40 region-specific changes in the abundance of a wide range of metabolites (including carbohydrates, amino acids, nucleotides, and fatty acids) in the livers of mice at two different infection times, which also revealed the heterogeneous metabolic characteristics of the liver egg granulomas of *S. japonicum*. In a chronic infection model with *S. japonicum,* oral treatment with praziquantel significantly alleviated most metabolic disorders, including fatty acid and pyrimidine metabolism. Surprisingly, Upase1, a key enzyme in uridine metabolism, was significantly upregulated 6 weeks after infection, and liver uridine levels were negatively correlated with the abundance of multiple lipid-associated metabolites. Further studies revealed that in vitro uridine supplementation inhibited the activation of LX-2 cells, restored the homeostasis of fatty acid metabolism through the peroxisome proliferator-activated receptor γ (PPARγ) pathway, and played an antifibrotic role. Our findings provide new insights into the molecular mechanisms of *S. japonicum*-induced liver fibrosis and the potential of targeting uridine metabolism in disease therapy.

## Author summary

In this study, we used AFADESI-MSI technology to investigate the variations in metabolite abundance in the liver during acute and chronic *S. japonicum* infection and the effects of PZQ treatment on the liver metabolism of schistosomiasis-infected mice. We aimed to identify and visualize alterations in liver-related metabolites at a spatially detailed metabolic level and map the distribution patterns of these metabolites within the egg granuloma region. The differentially abundant metabolites and metabolic pathways identified were compared and analyzed, It was found that the disturbance of uridine metabolism was a significant metabolic pathway in the course of infection and was related to lipid metabolism. In addition, focusing on the mechanism of lipid reprogramming and cell activation induced by the disturbance of uridine metabolism in hepatic stellate cells. This study not only increases our understanding of the metabolic disorders in the liver during the development of schistosomiasis-related liver disease but also highlights the importance of the disturbance of uridine metabolism in the development of liver fibrosis and pinpoints important candidate metabolites for future drug development.

## Introduction

Schistosomiasis is a parasitic disease that is widely prevalent in tropical and subtropical regions and poses a serious health risk to humans [1,2]. The World Health Organization has estimated that approximately 779 million people are at risk of schistosomiasis infection worldwide and that more than 250 million people are infected with schistosomiasis [3,4]. The main schistosomes that infect humans are *Schistosoma mansoni*, *Schistosoma japonicum (S. japonicum)*, and *Schistosoma haematobium*, of which *S. japonicum* is prevalent mainly in China [5,6]. The core damage caused by *S. japonicum* infection is liver damage and persistent granulomatous reactions caused by eggs in the liver. The chronic development of this disease leads to liver fibrosis and its complications, which are also important causes of death in schistosomiasis patients [7]. However, the precise molecular mechanisms underlying liver fibrosis caused by *S. japonicum* remain unclear within the scientific community. Additionally, the development of therapeutic drugs in clinical settings relies on a deeper understanding of these mechanisms.

Metabolomics is the comprehensive analysis of small-molecule metabolites present in living organisms under specific conditions and is a powerful tool for identifying new drug targets, discovering biomarkers, surveilling disease, and investigating disease pathogenesis [8]. At present, metabolomics methods have been widely used to find new therapeutic targets for parasitic infections [9]. Nonetheless, owing to technical constraints, most current metabolomics investigations of diseases, including schistosomiasis, have involved the use of primarily on serum and urine samples [10]. A common limitation in the metabolic analysis of complex biological samples is the inability to consider the intercellular heterogeneity within organs or tissues. Consequently, achieving comprehensive visualization of systemic metabolic reprogramming and its interactions with liver diseases remains a formidable challenge [11]. The advent of mass spectrometry imaging (MSI) technology has introduced novel possibilities for studying organ-specific heterogeneity in diseased tissues and the spatial distribution patterns of metabolites [12]. Spatial metabolomics, which is based on MSI, facilitates the onsite screening of metabolic biomarkers associated with the development of liver lesions. This approach facilitates a detailed depiction of the metabolic landscape within liver lesion sites and their surrounding microenvironments, offering a distinct advantage in exploring disease mechanisms [13]. Therefore, the use of spatial metabolomics approaches and in-depth pathway analysis can improve our understanding of disease progression and drug action modes.

Praziquantel (PZQ) is a broad-spectrum antiparasitic drug and is the first choice for the treatment of schistosomiasis [14]. Praziquantel is believed to have three unique pharmacological effects on *Schistosoma*, namely, stimulation of worm motor activity, spastic contraction of muscle tissue, and the formation of outer skin vesicles [15]. These actions expose the parasite's surface antigens, which enhances the ability of the host immune system to attack them. The effectiveness of PZQ is diminished in mice with depleted T or B cells, highlighting the drug's reliance on the host immune response for optimal efficacy. Additionally, PZQ reduces inflammation around eggs and prevents liver fibrosis. However, the precise mechanisms through which PZQ combats schistosomiasis remain unclear. Schistosomes rely on host nutrients, causing metabolic changes that benefit parasites. Disruption of nutrient intake can harm parasites and mitigate the associated metabolic changes in the host. Understanding liver metabolism following schistosome infection is crucial for identifying key factors involved in nutrient exchange between parasites and hosts. Investigating liver metabolic pathways related to PZQ treatment may reveal new targets for the development of antischistosomiasis drugs.

Uridine is a type of uracil nucleoside that plays a vital role in RNA and DNA biosynthesis, glycogen deposition, protein and lipid glycosylation, and body temperature and circadian rhythm maintenance and is closely associated with many metabolic diseases [16]. The balance of uridine in the body is regulated by uridine phosphorylase 1 (Upase1), which catalyzes the conversion of uridine into uracil [17]. A series of studies have shown that the disruption of uridine homeostasis is related to the occurrence and development of diabetes, neurodegeneration, fatty liver, obesity, and other diseases [16]. A study on liver disease revealed that increasing liver uridine levels through CPBMF 65 (a uridine phosphorylase 1 inhibitor) mitigated CCl4-induced liver fibrosis in mice [18]. In addition, studies have demonstrated that there is crosstalk between uridine metabolism and lipid metabolism [19], and uridine supplementation can prevent tamoxifen-induced nonalcoholic fatty liver disease (NAFLD) and stimulate liver phospholipid biosynthesis [20]. Nevertheless, the role of uridine metabolism in schistosomiasis-induced liver fibrosis remains unknown.

In this study, we used air-flow-assisted desorption electrospray ionization-mass spectrometric imaging (AFADESI-MSI) technology to investigate the variations in metabolite abundance in the liver during acute and chronic *S. japonicum* infection and the effects of PZQ treatment on the liver metabolism of schistosomiasis-infected mice. We aimed to identify and visualize alterations in liver-related metabolites at a spatially detailed metabolic level and map the distribution patterns of these metabolites within the egg granuloma region. The differentially abundant metabolites and metabolic pathways identified were compared and analyzed, focusing on the mechanism of lipid reprogramming and cell activation induced by the disturbance of uridine metabolism in hepatic stellate cells. This study not only increases our understanding of the metabolic disorders in the liver during the development of schistosomiasis-related liver disease but also highlights the importance of the disturbance of uridine metabolism in the development of liver fibrosis and pinpoints important candidate metabolites for future drug development.

## Results

### Survival and liver pathological analyses of the mice

**Fig 1:**
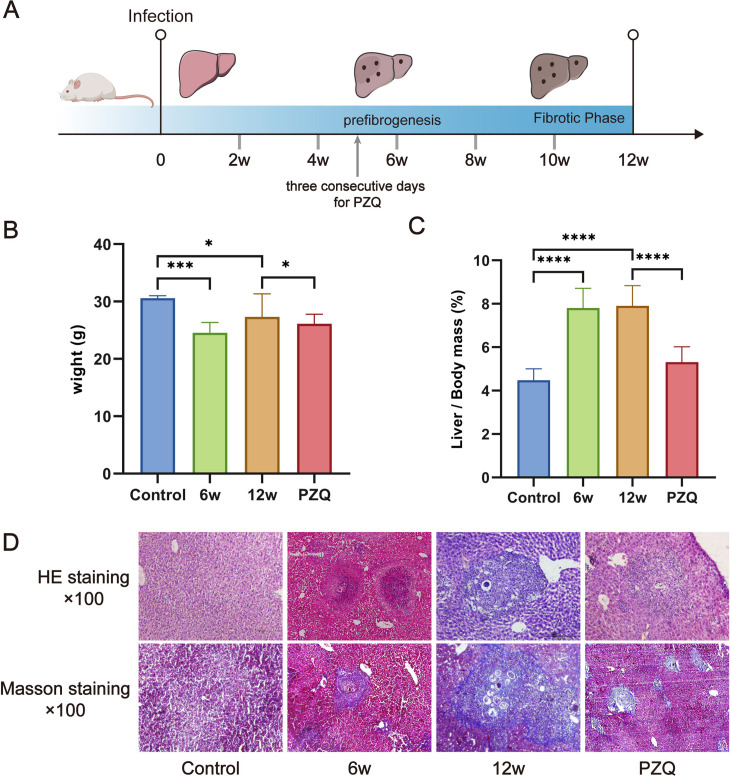
Survival and liver pathology analyses of mice infected with *S. japonicum.* (A) Schematic diagram of the experimental design of the mouse model infected with *S. japonicum*. The illustration in Fig 1A was drawn by hand using Adobe Illustrator 2021 software. (B) Histogram of the liver weights of the mice. (C) Liver/body weight ratios of the mice. (D) HE and Masson staining of the liver tissue of the mice (×100). The data are presented as the mean ± SD. Statistical significance is shown as ^*^P < 0.05, ^***^P < 0.001, and ^****^P < 0.0001, n = 5.

*S. japonicum* infection predominantly leads to liver damage in the host. We constructed mouse models of acute and chronic *S. japonicum* infection and treated the model mice with PZQ (Fig 1A). Compared with those in the uninfected group, the body weights, activity, and mental states of the mice in the S*. japonicum*-infected group were significantly lower at 6 and 12 weeks (Fig 1B). Moreover, the liver volume and weight increased significantly, and the liver index increased significantly (Fig 1C). The liver samples were subsequently harvested for histopathology. HE staining revealed obvious infiltration of immune cells and egg granulomas in the livers of infected mice. The area of egg granulomas increased significantly in the 12w group (Fig 1D). Masson staining revealed obvious collagen fibrotic deposition around egg granulomas, suggesting that *S. japonicum* infection may lead to liver injury and liver fibrosis with the chronic development of infection (Fig 1D). In addition, PZQ treatment significantly ameliorated related symptoms in mice. Compared with those of the infected group, the mental state of the infected group improved, the liver index decreased, and pathological liver injury was alleviated (Fig 1B–1D).

### Spatial metabolomics reveals liver metabolic variations in different stages of *S. japonicum* infection in mice

**Fig 2:**
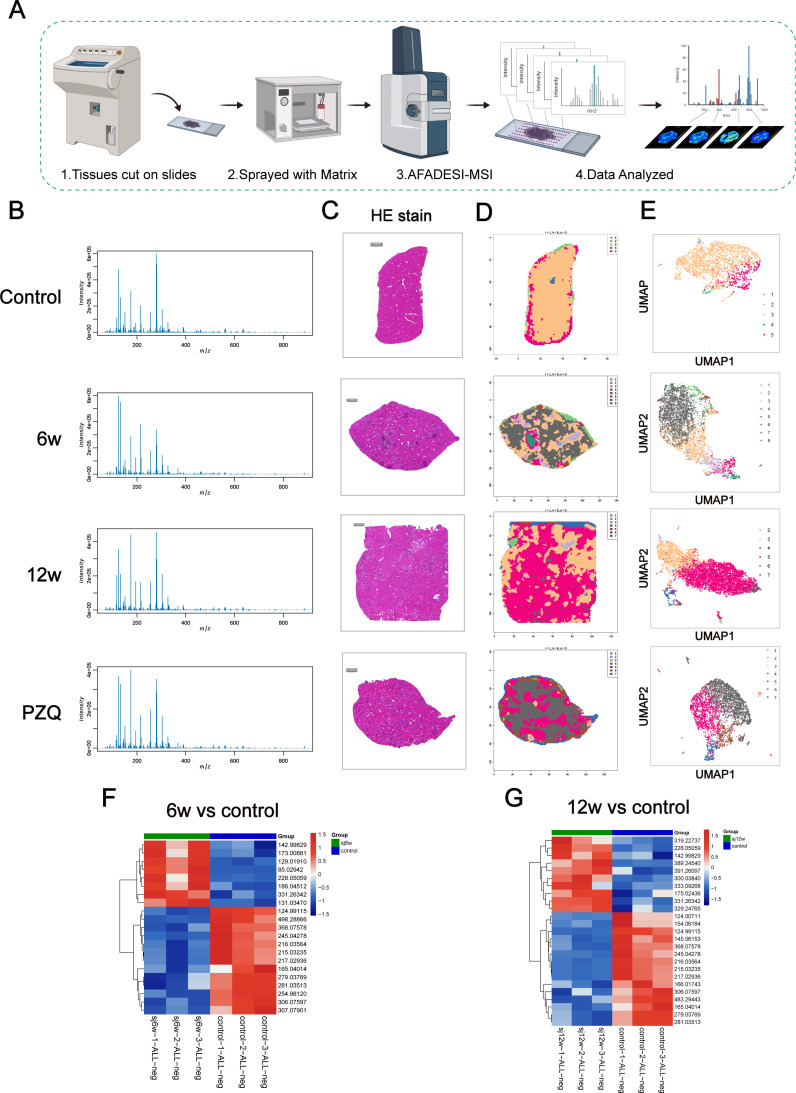
AFADESI-MSI imaging of liver metabolites. (A) Schematic of the workflow for mass spectrometry imaging. The illustration in Fig 2A was created with BioRender.com. (B) Representative mass spectra of liver samples from each group. A was created with BioRender.com. (C) HE staining of an immediate adjacent section was used for AFADESI-MSI for histopathological assessment. Scale bar: 1000 μm. (D) Imaging analysis of specific metabolites in different liver tissues. (E) UMAP analysis of specific metabolites in different liver tissues. (F) Clustering heatmaps of differentially expressed metabolites between different samples in the control and 6w groups (n = 3). (G) Clustering heatmaps of differentially expressed metabolites between different samples in the control and 12w groups (n = 3).

Liver injury is a prominent pathology following *Schistosoma* infection. Mature female worms begin laying eggs around 4 to 5 weeks post infection, with eggs deposited in the liver at approximately 6 weeks, leading to the formation of acute egg granulomas and subsequent acute liver injury. Here, we investigated hepatic metabolic changes during the early stage of egg deposition (6 w) via spatial metabolomics techniques to better understand the relationships between hepatic metabolites and disease progression (Fig 2A). We initiated the analysis by selecting a representative mass spectrogram for comparison with the control group. This comparison revealed differences in the peak distributions of metabolites between the two groups, indicating significant variation in substance distribution (Fig 2B). Principal Component Analysis (PCA) was employed to analyze the spatial metabolomics results of liver tissues from the mice in the 6-week infected group and the control group. The results indicated that the samples from both groups fell within the 95% confidence interval, with no abnormal samples observed (S1A Fig). The results demonstrated a significant separation between the two groups of data on the first principal component, emphasizing the substantial metabolic differences (S1B Fig).

**Fig 3:**
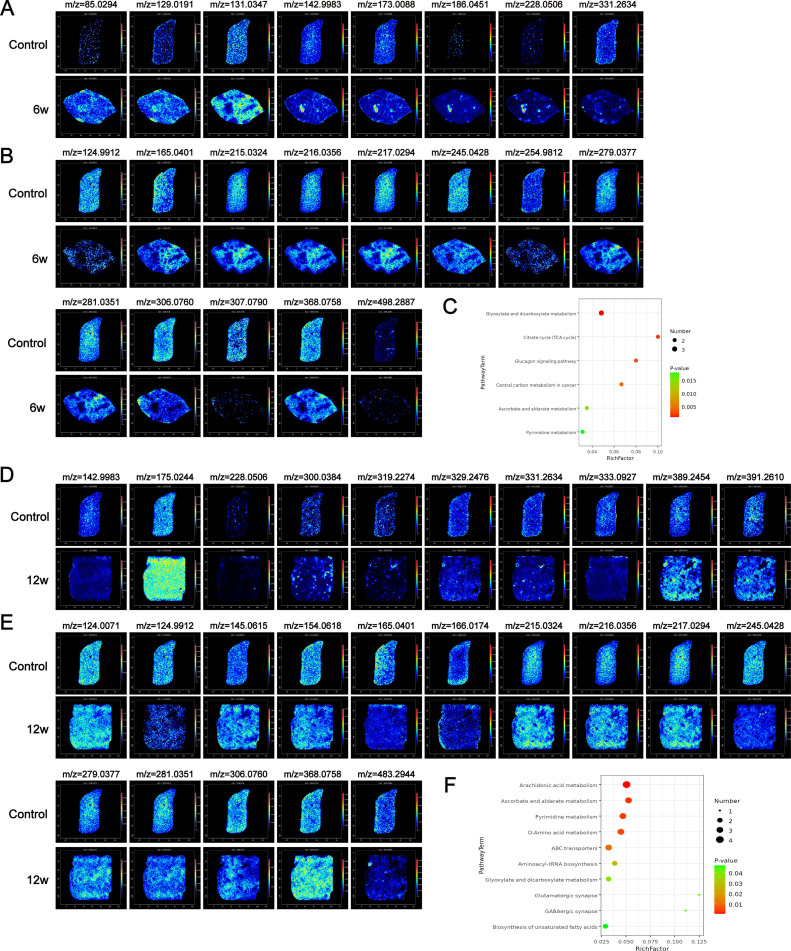
In situ visualization of crucial metabolites and metabolic pathways in the 6w vs. control or 12w vs. control groups. (A) MS images of upregulated ions in the 6w vs. control comparisons. (B) MS images of downregulated ions in the 6w vs. control comparisons. (C) The significantly (*P* < 0.05) enriched pathways for differentially abundant metabolites in the 6w vs. control comparisons. (D) MS images of upregulated ions in the 12w vs. control comparison. (E) MS images of downregulated ions in the 12w vs. control comparison. (F) The significantly (*P* < 0.05) enriched pathways for differentially abundant metabolites in the 12w vs. control comparisons.

To enhance the visual representation of the relationships between samples and the differences in metabolite expression across various samples, hierarchical clustering was conducted on all differentially abundant metabolites. The resulting heatmap utilized a color gradient from blue to red to depict the abundance of metabolites, with redder shades indicating a greater abundance (Fig 2F). The clustering diagrams distinctly manifested substantial regionalization in the distribution of liver metabolites following infection (Fig 2D), a finding that was further corroborated by UMAP analysis (Fig 2E). In addition, the comparison of HE staining results showed that the metabolic distribution of egg granuloma tissue was significantly different from the surrounding tissue (Fig 2C). Upon further analysis, compared with those in the control group, the liver tissues of the mice in the 6-week infection group presented 21 potential differential metabolites under negative ion mode, with 8 metabolites that increased in abundance and 13 that decreased in abundance (S1C Fig and S2 Table). Moreover, we performed mass spectrometry imaging of these differentially abundant metabolites (Fig 3A and 3B). KEGG pathway enrichment analysis was subsequently conducted for all differentially abundant metabolites, resulting in the identification of 16 differential metabolic pathways (S1G Fig). Among these pathways, 6 were found to be significantly impacted (Fig 3C and S3 Table). These pathways include glyoxylate and dicarboxylate metabolism, the citrate cycle (TCA cycle), the glucagon signaling pathway, central carbon metabolism in cancer, and ascorbate and aldarate metabolism.

The progression of chronic egg granuloma formation plays a pivotal role in inducing liver fibrosis in schistosomiasis. We also analyzed liver metabolite changes in mice during the chronic infection period, specifically at 12 weeks postinfection. The data quality was assessed via mass spectrometry (Fig 2B), principal component analysis (PCA) (S1D Fig), and OPLS-DA modeling (S1E Fig). Compared with the uninfected group, these analyses revealed significant differences between the two groups, highlighting notable variations in liver metabolite abundance (Fig 2G). UMAP analysis effectively clustered the metabolites into distinct groups (Fig 2D and 2E). Further analysis revealed that, in contrast with those in the control group, the liver tissues of the mice in the 12-week infection group presented 25 potential differential metabolites in negative ion mode. Among these, the abundance of 10 metabolites increased, whereas the abundance of 15 decreased (S1F Fig and S4 Table). The mass spectrograms corresponding to these differentially abundant metabolites are presented in Fig 3D and 3E. Moreover, KEGG pathway enrichment analysis was conducted for all differentially abundant metabolites, leading to the identification of several differential metabolic pathways (S1H Fig). Among these pathways, 10 were significantly enriched (Fig 3F and S5 Table). These pathways included arachidonic acid metabolism, ascorbate and aldarate metabolism, pyrimidine metabolism, D-amino acid metabolism, and ABC transporters, among others.

### Spatial metabolomics reveals the metabolic heterogeneity of hepatic egg granulomas in *S. japonicum*-infected mice

**Fig 4:**
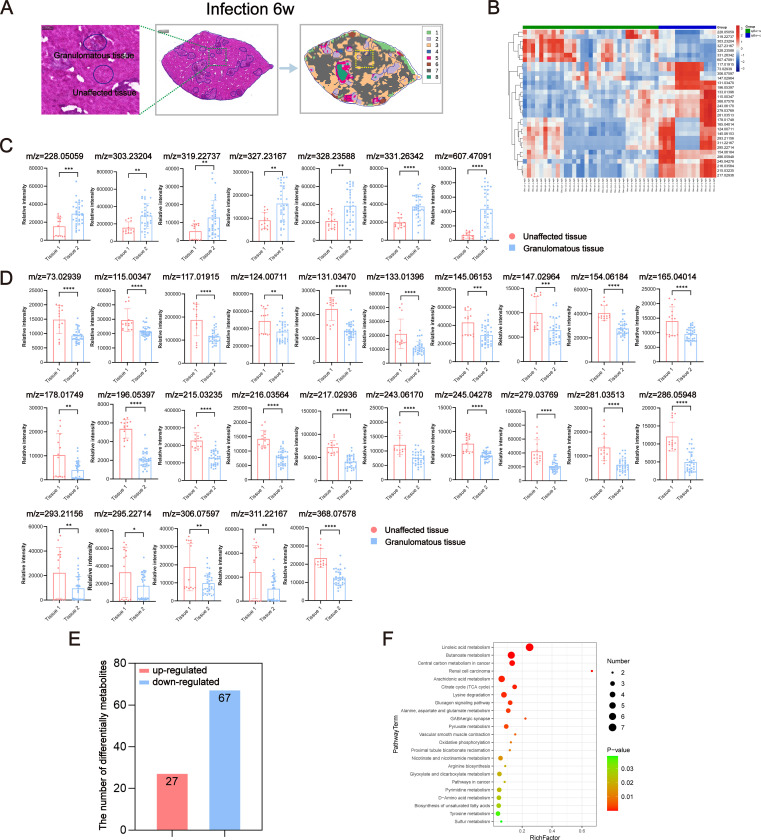
Discriminating metabolites were obtained through AFADESI-MSI analysis of the granulomatous tissue and unaffected tissue of 6w mice infected with *S. japonicum.* (A) Schematic diagram of the extraction and analysis of local metabolites from liver tissue at 6 weeks after infection. (B) Heatmap of spatially resolved metabolomics data. (C) Upregulation of ions in different regions in the granulomatous tissue vs. unaffected tissue comparisons. (D) Downregulation of ions in different regions in the granulomatous tissue vs. unaffected tissue comparisons. (E) Statistical map of the number of differentially abundant metabolites in the granulomatous tissue and unaffected tissue. (F) The significantly (*P* < 0.05) enriched pathways for differentially abundant metabolites in the granulomatous tissue vs. unaffected tissue. The data are presented as the mean ± SD. Statistical significance is shown as ^*^P < 0.05, ^**^P < 0.01, ^***^P < 0.001, and ^****^P < 0.0001.

**Fig 5:**
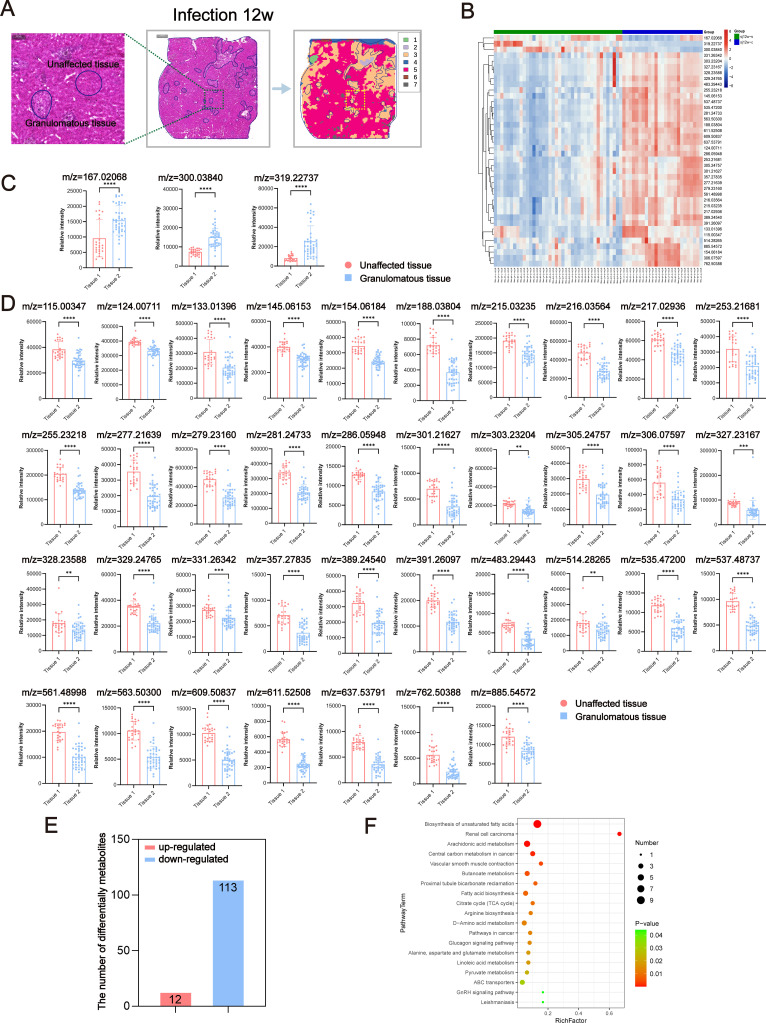
Differentially abundant metabolites were obtained through AFADESI-MSI analysis of the granulomatous tissue and unaffected tissue of 12w mice infected with *S. japonicum.* (A) Schematic diagram of the extraction and analysis of local metabolites from liver tissue at 12 weeks after infection. (B) Heatmap of spatially resolved metabolomics data. (C) Upregulation of ions in different regions in the granulomatous tissue vs. unaffected tissue comparisons. (D) Downregulation of ions in different regions in the granulomatous tissue vs. unaffected tissue comparisons. (E) Statistical map of the number of differentially abundant metabolites in the granulomatous tissue and unaffected tissue. (F) The significantly (*P* < 0.05) enriched pathways for differentially abundant metabolites in the granulomatous tissue vs. unaffected tissue. The data are presented as the mean ± SD. Statistical significance is shown as ^**^P < 0.01, ^***^P < 0.001, and ^****^P < 0.0001.

*S. japonicum* infection is characterized by chronic liver injury, which includes the formation of liver egg granulomas. Metabolic interactions occurring within these granulomas and between granulomas and surrounding normal cells, such as immune cells and stromal cells, have a profound impact on the progression of hepatic injury and the immune response in schistosomiasis. To better understand the metabolic heterogeneity within hepatic egg granulomas, we divided the whole liver of postinfected mice into two tissue regions: granulomatous tissue and unaffected tissue (Figs 4A and 5A). We subsequently imported HE images with labeled sampling points into the AFADESI-MSI software MSiReader program for image fusion and spatial matching. Subsequently, we extracted region-specific in situ AFADESI-MSI spectra in accordance with the labeled sampling points in the HE images.

The quality of the extracted data was rigorously evaluated via mass spectrometry (S2A and S2E Fig), PCA (S2B and S2F Fig), and OPLS-DA model analysis (S2C and S2G Fig). The results consistently demonstrated significant differences in liver metabolic profiles between granulomatous tissue and unaffected tissue. Furthermore, the liver tissue metabolites of mice infected with *S. japonicum* exhibited a significant regional distribution, a finding that was also corroborated by UMAP analysis, as described in an earlier section of the results. Through an analysis of liver metabolites in mice infected for 6 weeks, we identified 32 potential differential metabolites in the egg granuloma tissues of the liver in negative ion mode. Among these, the abundance of 7 metabolites was increased, whereas the abundance of 25 were decreased (S2D Fig and S6 Table). The abundance of individual ions in the samples is depicted via a heatmap (Fig 4B). A single differential ion may correspond to multiple potentially differential metabolites (Fig 4E). S6 Table provides details about the ions corresponding to these differentially abundant metabolites, and the mass spectrograms of the ions are displayed in S3A Fig. Fig 4C and 4D depict the relative expression intensity of each ion. Furthermore, we conducted KEGG pathway enrichment analysis for all differentially abundant metabolites, resulting in the identification of 62 enriched metabolic pathways (S3B Fig). These pathways encompass various aspects of metabolism, including glucose metabolism, lipid metabolism, amino acid metabolism and nucleic acid metabolism. Notably, significant differences were observed in 23 metabolic pathways, including pathways such as linoleic acid metabolism, butanoate metabolism, central carbon metabolism in cancer, renal cell carcinoma, and arachidonic acid metabolism (Fig 4F and S7 Table).

In the analysis of liver metabolites from mice infected for 12 weeks, compared with unaffected tissues, we identified 40 potential differential metabolites in the liver egg granuloma tissues under negative ion mode. Among these, the abundance of 3 metabolites were increased, whereas the abundance of 37 were decreased (S2H Fig and S8 Table). The expression of individual ions in the samples is depicted via a heatmap (Fig 5B). A single differential ion may correspond to multiple potentially differential metabolites (Fig 5E). S8 Table provides details about the ions corresponding to these differentially abundant metabolites, and the mass spectrograms of the ions are displayed in S4A Fig. Fig 5C and 5D depict the relative expression intensity of each ion. Additionally, we conducted KEGG pathway enrichment analysis for all differentially abundant metabolites, resulting in the identification of 60 enriched metabolic pathways (S4B Fig). These pathways encompass various aspects of metabolism, including glucose metabolism, lipid metabolism, and amino acid metabolism. Notably, KEGG pathway analysis revealed 19 pathways that were significantly enriched in the differentially expressed metabolites, including biosynthesis of unsaturated fatty acids, renal cell carcinoma, arachidonic acid metabolism, central carbon metabolism in cancer, and vascular smooth muscle contraction (Fig 5F and S9 Table).

### Spatial metabolomics reveals changes in liver metabolism in mice infected with *S. japonicum* and treated with PZQ

**Fig 6:**
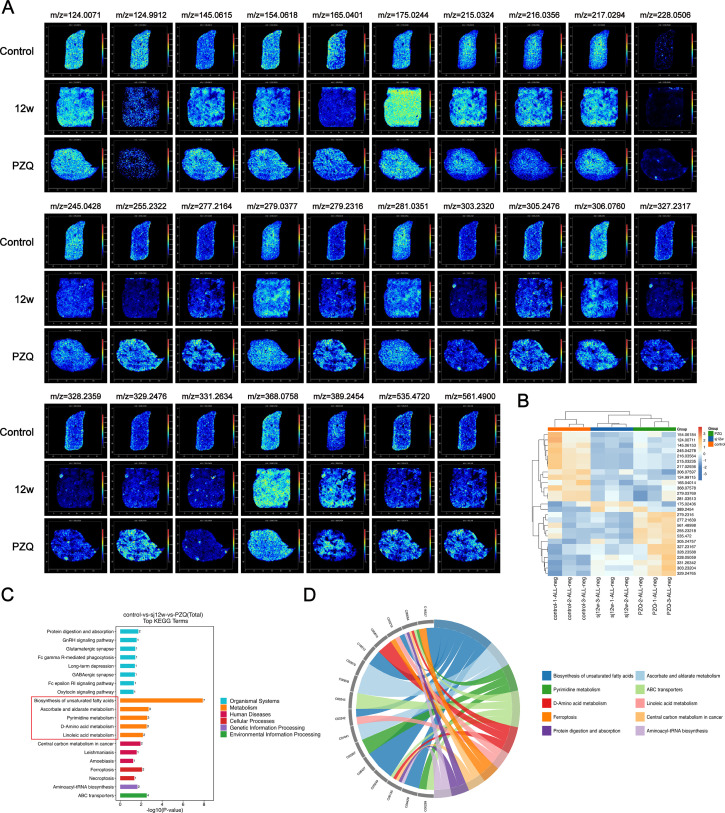
Metabolic regulatory effects of praziquantel on liver injury in mice infection with *Schistosomiasis japonica.* (A) MS images of situ visualization of crucial differentially abundant metabolites. (B) Heatmap of spatially resolved metabolomics data. (C) The top 20 enriched pathways for differentially abundant metabolites from KEGG. (D) The top 10 enriched pathways for differentially abundant metabolites from Reactome.

Praziquantel (PZQ) is acknowledged as the sole effective medication for schistosomiasis treatment. Our pathological findings indicate that PZQ treatment markedly suppresses the liver egg–granuloma reaction and impedes liver fibrosis progression. The significance of metabolic reprogramming in disease development and drug target screening has attracted increasing attention. Consequently, we further investigated the spatial distribution of liver metabolites linked to PZQ therapy via mass spectrometry imaging. Initially, data quality was assessed via multivariate statistical analysis, extending beyond principal component analysis (S5 Fig). The results revealed substantial disparities in liver metabolism characteristics among the mice treated with PZQ. In negative ion mode, 27 ions exhibited significant differences. Compared with those in the uninfected group, the expression intensities of these ions were either upregulated or downregulated following infection, with varying degrees of reversal observed after PZQ treatment (Fig 6A and 6B). Subsequently, 20 regions were randomly selected from each sample, and one-way ANOVA was employed to analyze the ion intensity in each region. This analysis revealed characteristic distribution differences in ion expression intensity among sample groups. A list of 27 distinct metabolites corresponding to significantly different ions is provided in Table 1. Subsequent pathway enrichment analysis of metabolites via KEGG revealed enrichment of 5 metabolic pathways with significant differences (Fig 6C). In addition, Reactome analysis revealed enrichment of multiple common metabolic pathways (Fig 6D). The biosynthesis of unsaturated fatty acids, ascorbate and aldarate metabolism, pyrimidine metabolism, D-amino acid metabolism, and linoleic acid metabolism suggest the potential involvement of metabolic pathways, such as unsaturated fatty acids, in the development of schistosomiasis-related liver disease and coordination in the antischistosomiasis process of PZQ.

**Table 1:** Metabolic regulatory effects of praziquantel on liver injury of schistosomiasis japonica.

m/z	Metabolites	VIP	Control	12w	PZQ	12w-Control	PZQ-12w
279.232^*^	N1,N11-Bis(ethyl)norspermine	6.32103	426905	375217	735160	↓	↑
124.007^*^	Taurine	6.17374	725802	356376	433007	↓	↑
215.032^*^	Sodium ferulate	5.15556	404823	161211	227179	↓	↑
255.232^*^	FA(16:0) Trimethyltridecanoic acid Isopalmitic acid Butyl dodecanoate Hexyl decanoate	3.88923	197270	175794	307345	↓	↑
175.024^*^	Ascorbic acid D-Glucurono-6,3-lactone 1,2,3-Propanetricarboxylic acid	3.87687	295380	428160	298469	↑	↓
303.232	FA(20:4) Cis-8,11,14,17-Eicosatetraenoic acid Mesterolone Copalic acid 7,13-Eperudien-15-oic acid	3.8392	123473	178743	270198	↑	↑
217.029^*^	Coumestan	2.90441	129180	51588.3	72640.6	↓	↑
327.232	FA(22:6) Neogrifolin Grifolin Retinol acetate	2.56014	57730.6	67848.9	115873	↑	↑
306.076	zaprinast	2.08856	69286.8	41581.4	27905.1	↓	↓
277.216^*^	FA(18:3) Calendic acid Punicic acid Linolenelaidic acid	2.04646	27597.1	27317.7	60408.9	↓	↑
145.062^*^	Alanylglycine L-Glutamine D-Glutamine	1.83887	67401.8	35202.5	47071.2	↓	↑
279.038	Uridine Pseudouridine	1.81271	70915.2	45738.2	42866.7	↓	↓
368.076	4-[4-(Quinolin-2-ylmethoxy)phenyl]sulfanylbenzoic Acid	1.7301	45460.4	23549.6	21987.5	↓	↓
329.248	22:5 FA(22:5) 4,8,12,15,19-Docosapentaenoic acid	1.69268	12027.5	27128.7	40275.5	↑	↑
245.043	Glycerophosphoglycerol	1.59156	35216.4	11027.2	17541.3	↑	↑
331.263	FA(22:4) 1-Hydroxy-1-phenyl-3-hexadecanone 3-Hydroxy-1-phenyl-1-hexadecanone Ethyl Arachidonate	1.54282	8443.66	23474.9	32248.2	↑	↑
561.49^*^	DG(33:1)	1.4199	21679	14480.9	35250.2	↓	↑
154.062^*^	L-Histidine	1.40789	49883.3	28128.3	36464.5	↓	↑
165.040^*^	Arabinonic acid Ribonic acid	1.39966	28062.3	11008.1	17865.1	↓	↑
216.036^*^	N,N-Bis(2-chloroethyl)aniline	1.31694	25598.2	9832.24	14044.2	↓	↑
389.245^*^	MG(18:2)	1.28627	8619.01	23943	19887.1	↑	↓
328.236	Carrageenan, potassium salt of	1.24681	13214.4	15603	26925.4	↑	↑
305.248	FA(20:3) 5,8,11-Eicosatrienoic acid; Sciadonic acid	1.22072	17462.1	24126.1	33415.7	↑	↑
535.472^*^	FAHFA(18:1(9Z)/6-O-16:0)	1.13959	12194.8	8764.94	21220.5	↓	↑
281.035	Tazobactam	1.04036	22909.1	14631.1	13636.9	↓	↓
228.051	4,5-seco-dopa	1.03428	596.344	7457.07	10747.7	↑	↑
124.991^*^	Methanesulfinic acid	1.01997	9924.43	1172.98	1931.52	↓	↑

* Reverse regulation of ions after praziquantel treatment.

### Uridine metabolism is a significantly affected metabolic pathway during *S. japonicum* infection

**Fig 7:**
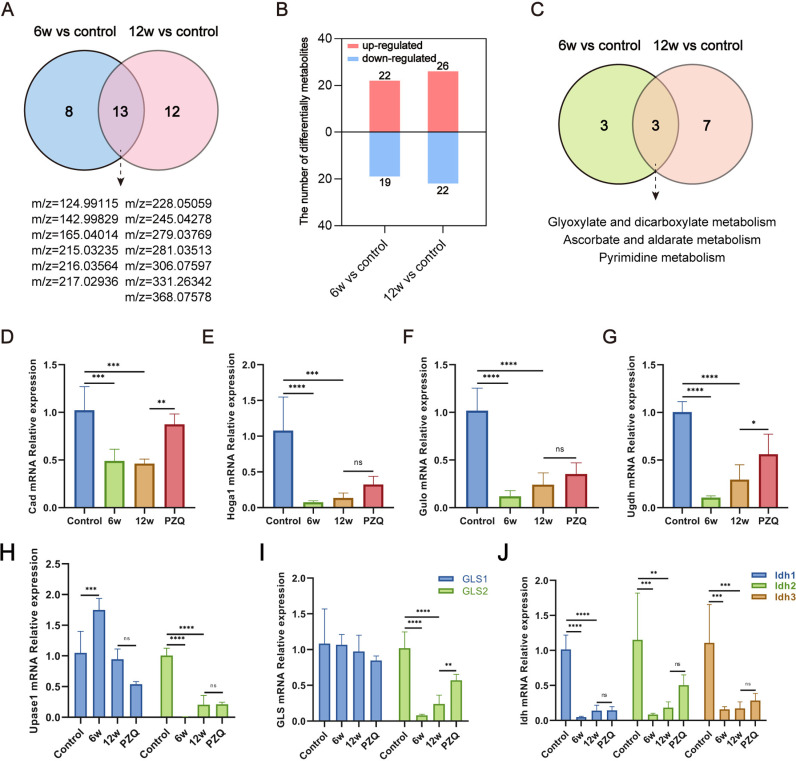
Uridine metabolism is significantly affected by metabolic pathways during *S. japonicum* infection in mice. (A) Venn diagrams displaying the common differentially abundant metabolites, 6w vs. control and 12w vs. control comparisons. (B) Statistical map of the number of potential differentially abundant metabolites, 6w vs. control and 12w vs. control comparisons. (C) Venn diagrams displaying the common metabolic pathways, 6w vs. control, and 12w vs. control comparisons. (D) Relative expression level of the rate-limiting enzyme CAD mRNA in the de novo synthesis pathway of uridine in the livers of mice. (E) Relative expression levels of HOGA1, a key enzyme in the glyoxylate metabolic pathway, in the livers of mice. (F) The relative expression level of GULO, a key enzyme in the ascorbic acid synthesis pathway, in the livers of the mice. (G) The relative expression level of UGDH mRNA, a key enzyme in the glucuronic acid metabolic pathway, in the livers of the mice. (H) The relative expression level of Upase, a key enzyme in the uridine metabolic pathway, in the livers of the mice. (I) The relative expression level of Upase, a key enzyme in the glutamine metabolic pathway, in the livers of the mice. (J) Relative expression levels of IDH, a key enzyme in the tricarboxylic acid cycle, in the livers of mice. At least three replicate samples were used for each experiment. The data are presented as the mean ± SD. Statistical significance is shown as * *P* < 0.05, ** *P* < 0.001, *** *P* < 0.001, **** *P* < 0.0001.

To gain deeper insights into the relationship between metabolic disturbances and the disease process, we conducted a more detailed analysis of the identified differentially abundant metabolites. Our investigation revealed that multiple metabolites and metabolic pathways were significantly impacted during both the early and chronic stages of infection (Fig 7A and 7B). Thirteen characteristic metabolites and 3 metabolic pathways related to disease development may be involved in the chronicity of schistosomiasis. The number and types of potential differential metabolites corresponding to the corresponding differential ions are listed in Table 2. This pathway mainly includes three metabolic pathways: glyoxylate and dicarboxylate metabolism, ascorbate and aldarate metabolism, and pyrimidine metabolism (Fig 7C). These findings suggest a close association between these three metabolic pathways and disease progression. Upon further examination of these three differential metabolic pathways in conjunction with the identified differentially abundant metabolites, we found that glutamine and uridine play central roles in connecting these differentially abundant metabolites. Furthermore, in a pathway enrichment analysis of differentially abundant metabolites in the livers of mice in the control and 6-week infection groups, we also identified the pathways associated with ascorbate and aldarate metabolism. This result corroborates our earlier studies on the metabolomics of serum samples [10]. Overall, this analysis highlights the importance of metabolites in the relationships that regulate the effects of disease processes. In addition to the identified differential metabolites, RT‒qPCR verification of related metabolic enzymes was performed. Surprisingly, we found that Upase1, a crucial enzyme of uridine metabolism, was significantly upregulated at 6 weeks of infection, whereas other metabolic enzymes were downregulated. HSCs activation is widely recognized as the central event in the development of schistosomiasis -related liver fibrosis. Consistently, we also observed disruptions in uridine metabolism within the HSCs cell line (S6A and S6B Fig). In addition, the expression of most metabolic enzymes could be reversed by PZQ treatment (Fig 7D–7J). These results suggest that the disturbance of uridine metabolism may play an important role in the occurrence and development of liver fibrosis in schistosomiasis.

**Table 2:** Summary of common differential metabolites between the infected group 6w and the infected group 12w, compared with the control group.

m/z	Formula	Metabolites	Control	6w	12w	6w vs. control	12w vs. control
124.9912	CH_4_O_2_S	Methanesulfinic acid	9924.43	877.89	1172.98	↓	↓
142.9983	C_5_H_6_O_6_	4-Hydroxy-2-oxoglutaric acid	16433.89	26874.26	23385.72	↑	↑
165.0401	C_5_H_10_O_6_	Arabinonic acid; Ribonic acid	28062.29	12994.06	11008.06	↓	↓
215.0324	C_10_H_9_NaO_4_	Sodium ferulate	404823.30	185036.42	161211.33	↓	↓
216.0356	C_10_H_13_Cl_2_N	N,N-Bis(2-chloroethyl)aniline	25598.22	11501.96	9832.24	↓	↓
217.0294	C_15_H_8_O_3_	Coumestan	129180.04	59021.64	51588.29	↓	↓
228.0506	C_9_H_11_NO_6_	4,5-seco-dopa	596.34	25528.18	7457.07	↑	↑
245.0428	C_6_H_15_O_8_P	Glycerophosphoglycerol	35216.44	6803.56	11027.21	↓	↓
279.0377	C_9_H_12_N_2_O_6_	Uridine; Pseudouridine	70915.19	34604.26	45738.17	↓	↓
281.0351	C_10_H_12_N_4_O_5_S	Tazobactam	22909.10	10812.06	14631.15	↓	↓
306.0760	C_13_H_13_N_5_O_2_	zaprinast	69286.77	11837.04	41581.41	↓	↓
331.2634	C_22_H_36_O_2_	FA(22:4); 1-Hydroxy-1-phenyl-3-hexadecanone; 3-Hydroxy-1-phenyl-1-hexadecanone; Ethyl Arachidonate	8443.66	26647.46	23474.91	↑	↑
368.0758	C_23_H_17_NO_3_S	4-[4-(Quinolin-2-ylmethoxy)phenyl]sulfanylbenzoic Acid	45460.39	19711.58	23549.65	↓	↓

### Uridine regulates fatty acid metabolic reprogramming and cell activation in LX-2 cells through the PPARγ pathway

**Fig 8:**
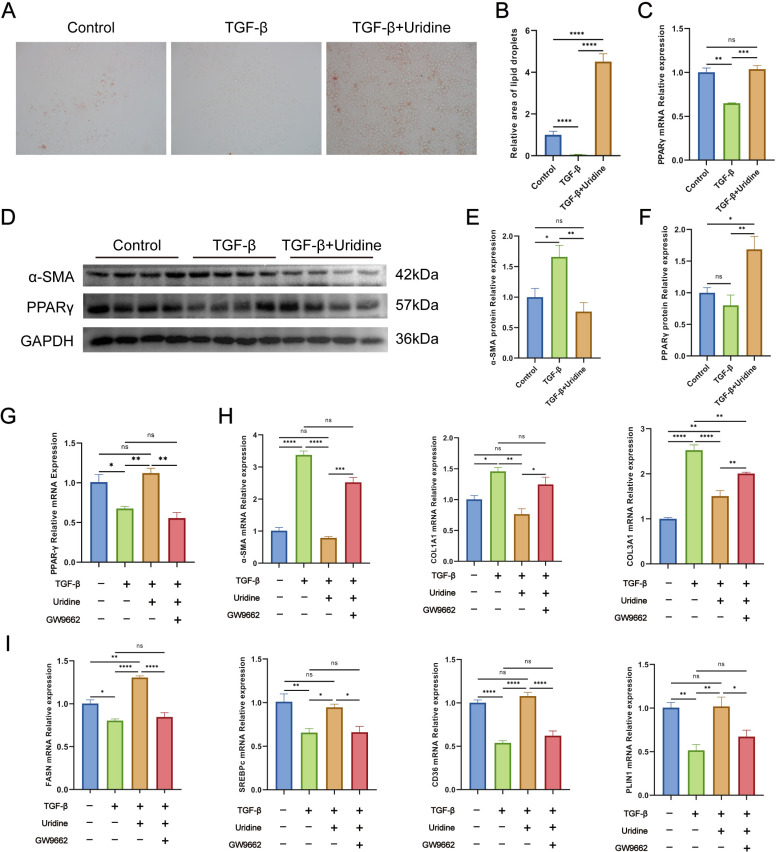
Uridine regulates lipid metabolic reprogramming and cell activation in LX-2 cells through the PPARγ pathway. (A) The effect of uridine intervention on lipid metabolism in activated LX-2 cells in vitro was addressed using via Oil Red O staining. (B) Statistical analysis of the lipid droplet area was performed using Oil Red O staining. (C) Effects of uridine supplementation in vitro on PPARγ gene expression in LX-2 cells. (D) Western blotting was used to detect the expression levels of PPARγ and α-SMA in LX-2 cells after uridine supplementation. (E) Effects of uridine supplementation in vitro on α-SMA protein expression in LX-2 cells. (F) Effects of uridine supplementation in vitro on PPARγ protein expression in LX-2 cells. (G)Effects of PPARγ inhibitor (WB9662) in vitro on PPARγ gene expression in LX-2 cells. (H) Effects of PPARγ pathway inhibitors on the activation of LX-2 cells after uridine intervention. (I) Effects of PPARγ pathway inhibitors on the expression of genes related to lipid metabolism in LX-2 cells after uridine intervention. At least three replicate samples were used for each experiment. The data are presented as the mean ± SD. Statistical significance is shown as * *P* < 0.05, ** *P* < 0.001, *** *P* < 0.001, **** *P* < 0.0001.

**Fig 9:**
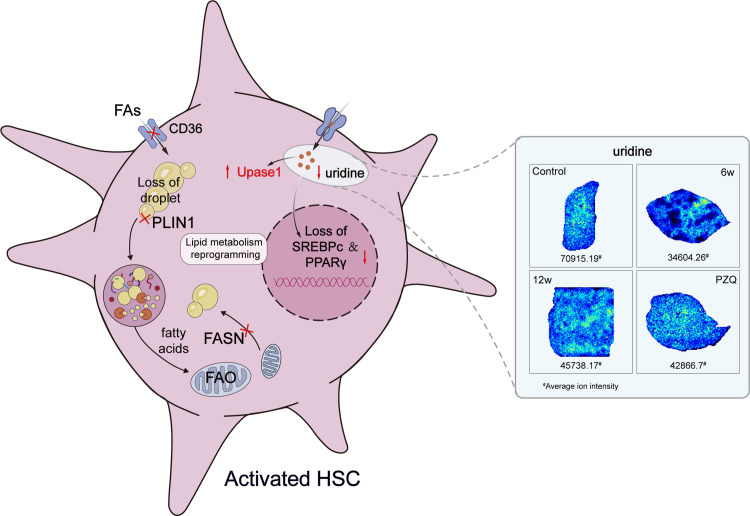
Summary of the mechanism by which uridine regulates lipid metabolism reprogramming in hepatic stellate cells. The illustration was drawn by hand using Adobe Illustrator 2021 software.

Upase1 is a key enzyme that catalyzes the metabolism of uridine. Upase1 was significantly upregulated in the livers of mice infected for 6 weeks. The catabolism of uridine in the liver was increased, and its content decreased. The cell experiments demonstrated that Benzylacyclouridine, a Upase1 inhibitor, effectively suppressed the expression of liver fibrosis-related genes in LX-2 cells *in vitro*, and reduced intracellular uridine levels (S6C–S6E Fig). This findings suggest that regulating of intracellular uridine levels could serve as a promising anti-fibrosis strategy. In addition, further correlation analysis of differentially abundant metabolites identified after PZQ treatment revealed a negative correlation between uridine and various lipid metabolism-related molecules (S5C Fig). The homeostasis of lipid metabolism in hepatic stellate cells is crucial for maintaining the quiescence of cells. To further investigate, we established a TGF-β-induced activation model of LX-2 cells *in vitro*, and examined the effects of uridine metabolism on lipid metabolism and hepatic stellate cells activation through uridine supplementation. Oil red O staining revealed that when activated LX-2 cells lost lipids, α-SMA protein expression was significantly upregulated. In contrast, uridine intervention inhibited cell activation, and the level of intracellular lipid drops recovered (Fig 8A–8F). PPARγ is considered a key regulatory factor involved in lipid metabolism. We found that PPARγ is significantly downregulated in activated LX-2 cells and upregulated by uridine intervention (Fig 8C, 8D and 8F). Further rescue experiments confirmed that uridine can regulate lipid metabolism homeostasis in LX-2 cells through the PPARγ signaling pathway, and the main mechanism may involve the upregulation of fatty acid transfer and the expression of key metabolic enzymes that play an antifibrotic role (Figs 8G–8I and 9).

## Discussion

In recent years, a growing number of studies have suggested that metabolic disorders involving carbohydrates, lipids, proteins, and hormones occur during the development of diseases, such as those of the lungs, liver, and kidneys, and that correcting these metabolic alterations may provide new strategies for treating disease [21]. The emergence of mass spectrometry imaging has provided researchers with a power tool to delve into the realm of fibro-metabolism [22]. In this study, we introduced an innovative approach by employing AFADESI-MSI to investigate the spatial distribution of liver metabolites in conjunction with the histological features characterizing the process of liver injury in *S. japonicum* infection. In addition, we conducted further experimental validation of key differentially abundant metabolites. In comparison with conventional metabolomics techniques, the primary advantage of AFADESI-MSI lies in its capacity to address biological questions pertaining to spatial variations with remarkable precision. Notably, this approach allows for the quantification and visual representation of alterations in liver metabolite profiles associated with the granulomatous tissues specific to *S. japonicum* infection. Such insights are important for advancing our understanding of the genesis and persistence of egg granuloma structures and the progression and treatment of liver fibrosis in the context of schistosomiasis.

The liver, as a pivotal metabolic hub within the body, plays a central role in schistosomiasis-associated pathology [23]. Previous investigations have highlighted the capacity of schistosome infection to disrupt host metabolic processes [10]. This phenomenon was further corroborated in our study through in situ mapping of liver metabolism. We successfully identified 41 and 48 differentially abundant metabolites in the early and chronic stages of schistosome infection, respectively. Notably, 18 of these metabolites exhibited substantial alterations and the same trend in both stages, underscoring their close association with disease progression (Fig 2G). The findings derived from KEGG pathway enrichment analysis revealed distinct impacts on nucleotide metabolism and carbohydrate metabolism in the liver in the early stages of infection. As infection progresses into the chronic phase, lipid metabolism, carbohydrate metabolism, nucleotide metabolism, and amino acid metabolism predominate. Notably, citric acid and isocitric acid were identified as unique differentially abundant metabolites in the early infection stage. These metabolites serve as crucial intermediaries within the TCA cycle and vital precursors for various biosynthetic processes. Their indispensability extends to numerous biological functions, including development, cellular homeostasis, and reproductive processes [24]. Schistosomes heavily rely on the host for essential nutrients, given their highly energy-intensive processes such as development, reproduction, and metabolism. These parasites avidly consume host-provided glucose and lipids to meet their metabolic needs [25]. The influence of schistosomiasis infection on lipid metabolism has been elucidated in prior studies [26]. Our study confirmed these observations, with significant interference detected in various fatty acid metabolism and synthesis pathways during the chronic infection phase. The most pronounced differences pertained to arachidonic acid metabolism and unsaturated fatty acid biosynthesis. The liver plays a vital role in the metabolic production of arachidonic acid, a substrate integral to the generation of numerous eicosanoid proinflammatory mediators [27]. Changes in arachidonic acid levels can mediate hepatocyte injury through diverse mechanisms, including neutrophil and macrophage activation, free radical production, and membrane lipid peroxidation [28]. The substantial alterations in various eicosanoic acids, represented by 15-HETE, in our results further support the notion of host liver damage induced by schistosome infection. Furthermore, our findings highlighted a significant downregulation of hepatic glutamine levels during chronic infection, establishing a pivotal link between multiple enriched metabolic pathways. Previous studies have extensively examined the bioactive roles of glutamine, particularly its ability to modulate immune cell functions [29]. For example, glutamine deprivation has been shown to inhibit T-cell proliferation and cytokine production, whereas T-cell activation is associated with increased glutamine metabolism [30,31]. Considering that chronic schistosomiasis encompasses a T-cell-mediated delayed-type hypersensitivity reaction [32], the decrease in glutamine levels suggests an elevated state of metabolic depletion, which is consistent with an immune-related disease state. Recent research has also implicated glutamine in liver injury [33,34], highlighting the need for further exploration of its role in schistosomiasis-associated liver fibrosis.

The chronicity of *Schistosomiasis japonica* infection is primarily attributed to the formation of egg granulomas within the liver [35]. Modern immunological studies have proposed that metabolic reprogramming plays a critical role in granuloma formation [36]. Our findings are consistent with this perspective, as they highlight the high metabolic heterogeneity exhibited by schistosome infection-induced granulomatous tissues. Owing to the structural complexity of egg granulomas, the identified differentially abundant metabolites and associated metabolic pathways exhibit a wide array of variations and complexities. Generally, dysregulation of linoleic acid metabolism, butanoate metabolism, and central carbon metabolism is prominently observed in the early stages of granuloma formation. During this phase, schistosome eggs tend to absorb essential nutrients from the host environment to support their development, encompassing the acquisition of energy resources and structural components required for synthesizing cell membranes [37,38]. Notably, our data revealed substantial alterations in various substances linked to the TCA cycle in proximity to early-stage eggs, possibly providing the energetic resources necessary for egg maturation. Additionally, since parasites are incapable of synthesizing lipids and rely on acquiring them from the surrounding environment [37], our results demonstrate a unique distribution of fatty acids within egg granulomatous tissue, offering further evidence of disrupted energy metabolism. During the acute inflammatory response induced by eggs, many immune cells accumulate around the eggs, indicating that metabolic reprogramming of egg granuloma tissues plays an important role in immune cell phenotype regulation. This phenomenon parallels observations made in the field of tumor immunology. In the chronic phase of infection, as eggs undergo starvation and calcification, the number of immune cells, particularly M2-type macrophages and type 2 helper T cells, increases [39,40]. Various studies have identified metabolic reprogramming as a defining characteristic of immune cells. M2-type macrophages, for example, tend to rely on mitochondrial oxidative phosphorylation metabolism to generate ATP, thereby supporting their survival and facilitating tissue repair. Their metabolic features include fatty acid oxidation, augmented arginine metabolic pathways, and activation of the tricarboxylic acid cycle [41,42]. Our study corroborates these observations, as we also detected reprogramming of these metabolic pathways in the liver with egg granulomas during chronic infection. These pathways involve the biosynthesis of unsaturated fatty acids, arachidonic acid metabolism, butanoate metabolism, linoleic acid metabolism, the citric acid cycle, central carbon metabolism, and arginine biosynthesis. Furthermore, metabolites have been recognized as regulators of T-cell phenotypes [43–45], with arachidonic acid, for example, exerting immunomodulatory effects by influencing the synthesis of prostaglandins, which primarily serve as anti-inflammatory molecules and inhibit the differentiation of Th1 cells [46]. Collectively, these changes in the metabolic profile tend to promote immune cell polarization, which is conducive to the persistence of granulomas. Remarkably, recent research has revealed that schistosome eggs can directly influence hepatic metabolic reprogramming independently of immune cells, particularly in the context of hepatic glycolipid metabolism [38]. Consequently, the specific mechanisms governing the establishment and maintenance of metabolic reprogramming within schistosome egg granulomas warrant further investigation. In conclusion, our data provide a robust dataset for explaining the course of *Schistosoma* infection-induced liver disease from a metabolic perspective.

*Schistosoma* survive by obtaining nutrients from the host, including proteins, glucose, lipids, and amino acids, through the digestive tract or the outer membrane [47,48]. Changes in liver metabolism during schistosomiasis infection have been discussed, and praziquantel, the drug of choice for the treatment of schistosomiasis, has been found to significantly alleviate most metabolic disorders in the liver after treatment. Lipid metabolism, including unsaturated fatty acid anabolism and arachidonic acid metabolism, was altered significantly. Many studies have shown that fatty acids are essential for *schistosoma* development and oviposition, mainly for maintaining oviposition and resisting host immune biosynthesis [49,50]. We found that multiple unsaturated fatty acids in the liver were reversed by PZQ treatment. Unsaturated fatty acids, which are believed to have anti-inflammatory effects, can promote the proliferation and differentiation of immune cells and enhance the function of the immune system [51,52]. These findings also suggest that PZQ therapy may regulate the differentiation of immune cells through the metabolic reprogramming of unsaturated fatty acids, thus playing an antischistosomiasis role, which indirectly provides a possible explanation for the poor efficacy of PZQ in immunodeficient mice. Notably, arachidonic acid (a fatty acid) has been considered an important target of PZQ in the treatment of schistosomiasis in previous studies [53]. In this study, we also observed significant changes in the liver metabolism of arachidonic acid before and after PZQ treatment. The parasite lacks raw materials to synthesize arachidonic acid and is dependent on the host. These findings also highlight the role of the arachidonic acid metabolic pathway in the treatment of PZQ. In addition, we also observed that carbohydrate, nucleotide, and amino acid metabolic pathways are related to PZQ therapy and that related metabolites may be involved in the process of PZQ resistance in schistosomiasis. Overall, we elucidated the potential effects of PZQ treatment on the metabolic interaction between *Schistosoma* and the host liver, and praziquantel may exert antischistosomiasis effects mainly by affecting liver fat metabolism and regulating parasite nutrient uptake and the immune microenvironment balance.

Notably, our study revealed a shared disruption in glyoxylate and dicarboxylate metabolism, ascorbate and aldarate metabolism, and pyrimidine metabolism during both the early and chronic phases of schistosomiasis. The metabolism of glyoxylate and dicarboxylate is closely linked to oxidation processes within organisms, contributing to the tricarboxylic acid (TCA) cycle that fuels energy metabolism. This pathway can perturb the activity of key enzymes, thereby resulting in abnormal levels of various reactants and products associated with the TCA cycle [54]. These findings offer additional insights into the influence of *Schistosoma* infection on energy metabolism. Within the ascorbate and aldarate metabolism pathway, a notable metabolite is D-glucuronic acid, which can undergo a series of enzymatic conversions to produce ascorbic acid. Ascorbic acid has been identified for its capacity to suppress inflammatory responses [55]. Although the human body lacks the enzyme system to synthesize ascorbic acid endogenously, our findings indirectly suggest that artificial supplementation with ascorbic acid may mitigate schistosomiasis-induced liver injury. The pyrimidine metabolism pathway is categorized under nucleotide metabolism and has garnered increased attention in relation to hepatic fibrosis in various studies [18,56]. Uridine, a pivotal component of this pathway, serves as a potential mediator of hepatoprotection [19]. Notably, our investigation revealed a substantial reduction in uridine levels within schistosome-infected liver tissues and revealed that Upase1 expression was significantly upregulated at 6 weeks of infection. This finding underscores the intricate interplay between pyrimidine metabolism and schistosomiasis-associated liver fibrosis.

The disturbance of uridine metabolism is associated with the progression of many diseases, and exogenous uridine supplementation is considered a potential strategy for the treatment of diseases [16]. We found that in vitro uridine supplementation significantly inhibited the activation of LX-2 cells, which was also consistent with the findings of several previous studies [57]. Spatial metabolomics analysis revealed that the content of uridine in the livers of schistosomiasis-infected mice was correlated with many lipid-related metabolites. We also observed that in vitro uridine supplementation increased the expression of lipid metabolism-related genes and the key pathway molecule PPARγ. PPARγ is a major transcriptional regulator of lipid formation and, together with other transcription factors, such as SREBP-1, induces adipocyte-specific gene expression [58]. In HSCs, normal expression of PPARγ may be necessary to maintain the quiescence of HSCs and is involved in the transdifferentiation of HSCs from lipose-storing quiescent cells to activated myofibrocytes. In quiescent HSCs, retinol-rich lipid droplets (LDs), which contain large amounts of lipids, are stored in the cytoplasm. Several studies have shown that the reduction in lipids in HSCs and the reduction in lipid-forming signals help maintain their activation state. The results of the cell oil red O staining confirmed these findings. Activated LX-2 cells lost lipids, and in vitro supplementation with uridine restored the number of intracellular lipid droplets. However, some studies have shown that uridine supplementation can reduce the formation of lipid droplets in hepatocytes and inhibit nonalcoholic fatty liver disease [59]. Overall, we identified the role of uridine metabolism in hepatic stellate cells, established the regulatory relationship between uridine metabolism and lipid metabolism homeostasis, and highlighted the influence of schistosomiasis infection on hepatic uridine metabolism and the potential role of uridine metabolism in the pathogenesis and treatment of liver fibrosis in schistosomiasis.

## Conclusions

In summary, we employed AFADESI-MSI to visualize the abundance of metabolites in the livers of mice infected with schistosomiasis. The identification of 41 and 48 metabolites associated with *S. japonicum*-induced liver injury at early and chronic infection stages, respectively, provides a comprehensive visual description of the overall metabolic heterogeneity of the liver and reveals the heterogeneous abundance of related metabolites in the egg granuloma region. In addition, PZQ treatment significantly reversed most metabolic disorders, especially those related to fatty acid metabolism and pyrimidine pathways. The key findings are that uridine metabolism affects cell activation by influencing fatty acid metabolism reprogramming in hepatic stellate cells and that uridine metabolism is involved in the progression of liver fibrosis. Our study provides key insights into the molecular pathology of schistosomiasis-related liver disease and the role of uridine metabolism in this disease, highlighting the potential of spatial metabolomics techniques in investigating therapeutic targets for the development of new liver disease treatments.

## Methods and methods

### Ethics statement

The study was approved by the Institutional Review Board (or Ethics Committee) of Jiangsu Institute of Parasitic Diseases (JIPD-2020-002).

### Experimental animals

A total of twenty female ICR mice (aged 6 weeks, weighing 20 ± 2 g) were purchased from Zhejiang Vital River Laboratory Animal Technology Co., Ltd. (license number: SCXK (Zhejiang) 2019-0001). The experimental animals were kept in the same environment at the Experimental Animal Center of Jiangsu Institute of Parasitic Diseases (license number: SYXK (Su) 2017-0050).

### Preparation of cercariae

The *Schistosoma japonicum* (Jiangsu strain) used in this study was preserved by the Jiangsu Institute of Parasitic Diseases. The cercariae were obtained from infected Oncomelania snails in our laboratory for use in animal experiments.

### Experimental groups and infection

A total of twenty mice were randomly divided into four groups of five mice each: an uninfected group (control), a 6-week infected group (6 w), a 12-week infected group (12 w), and the PZQ chemotherapy group (PZQ). They were given free access to food and water and were acclimated for 1–2 weeks before infection. The mice in the infected groups were infected with 15±2 cercariae per mouse via abdominal skin exposure [60]. At the fifth week postinfection, PZQ was dissolved in sodium carboxymethyl cellulose and administered to 5 mice by gavage at 150 mg/kg/day for three consecutive days to constitute the PZQ chemotherapy group. The survival status of the mice was periodically observed throughout the study.

### Cell culture

LX-2 cells, which were previously stored in our laboratory, were utilized for this study. The cells were reconstituted from a tube in liquid nitrogen, resuspended in DMEM (HyClone, SH3002201) containing 10% fetal bovine serum (Gibco, 10270106) and 1% penicillin-sulfur streptomycin, transferred to culture flasks, and incubated at 37 °C in an incubator containing 5% CO_2_. The cells were harvested when they reached 80% confluence, and other cell experiments were performed.

### Cell experimental grouping and treatment

LX-2 cells were cultured to 80% confluence, after which the cells were collected and seeded onto plates. After the cells attached to the bottom of the wells, activation of the LX-2 cells was induced by treatment with 25 ng/ml TGF-β (Absin; China) for 12 hours, and then, the complete medium containing 1 mg/ml uridine was replaced and cultured for 24 hours, after which the cells were collected for subsequent index detection. In the rescue experiment, after LX-2 cells were activated, the complete medium containing 1 mg/ml uridine (Beyotime, China) and 10 µg/ml PPARγ inhibitor (GW9662, Beyotime, China) was replaced, the mixture was cultured for 24 hours, and the cells were collected for subsequent index detection.

### Oil red O staining of cells

The oil red O dye storage solution and working solution were prepared in advance. The cell medium was discarded, the cells were washed with PBS twice, paraformaldehyde was added, and the cells were fixed at room temperature for 30 min. The fixing solution was discarded, the cells were washed with ddH_2_O twice and incubated in 60% isopropyl alcohol for 5 min. The alcohol was discarded, a previously prepared oil red O working solution was added, and the cells were incubated at 37 °C for 15 min. The solution was discarded, the cells were rinsed with 60% isopropyl alcohol until the interstitium was clear, and then the cells were washed with ddH_2_O until there was no excess dye. The cells were examined under a microscope, and pictures were taken.

### The mRNA expression levels of the genes were assessed via fluorescence quantitative PCR

Total RNA from cells or tissues was extracted with RNA-easy Isolation Reagent (Vazyme, R701), and the RNA purity (OD_260_/OD_280_ ratio in the range of 1.8–2.2) was verified with a NanoDrop instrument. cDNA was obtained through reverse transcription with a kit (Vazyme, R323-01). RNA levels were assessed with a LightCycler 480 instrument with SYBR qPCR Master Mix (Vazyme, Q711-02) and the indicated primers (S1 Table). After the threshold cycle (Ct) was obtained, the relative expression of the target gene was calculated via the 2^-ΔΔCt^ method with β-actin as the internal reference.

The primers were synthesized by Shanghai Bioengineering Co., Ltd., and the relevant primer sequences are shown in S1 Table.

### Western blot assays

Total protein was extracted from the cells, and the protein concentrations were determined via the BCA method. Protein samples were mixed with 6× SDS protein loading buffer at a ratio of 5:1 and boiled for 10 min at 95–100 °C. Each well was loaded with 30 μg of protein and subjected to SDS‒PAGE. The proteins were transferred to a PVDF membrane, which was blocked with 5% skim milk powder for 1–2 h. Primary antibodies (specific for α-SMA, Affinity; PPARγ, Proteintech, ab16502; GAPDH, Absin, abs132004) were added, and the membrane was incubated overnight at 4 °C. The blots were then washed three times for 10 min with TBST. The secondary antibodies were added and incubated for 1–2 h on a shaker at room temperature. After extensive washing, the protein bands were visualized with a Bio-Rad ChemiDoc XRS+ imaging system and quantified through densitometry via ImageJ software.

### Histopathological examination of liver samples

Small pieces of liver tissue from the right lobe of each mouse were fixed in a 4% paraformaldehyde solution for 24 hours. After gradient dehydration, transparency, wax immersion, embedding, sectioning, drying, dewaxing, rehydration, HE staining (Biosharp, BL700B), and Masson staining (Solarbio, G1340), the sections were placed on slides and coverslipped, observed under a light microscope, and photographed.

### AFADESI-MSI detection of related metabolites

#### Liver tissue processing.

Tissue samples fixed in embedding gel (Cryo-gel frozen section embedding agent) were removed from the −80 °C ultralow temperature refrigerator, thawed overnight in a −20 °C refrigerator, and sectioned (10 μm thickness) using a freezing microtome (Leica CM 1950, Leica Microsystem, Germany). The sections were immobilized on positive charge desorption plates (Thermo Fisher Scientific, Waltham, USA) and stored in an ultralow temperature refrigerator at −80 °C for subsequent imaging analysis.

#### Mass spectrometry imaging data acquisition.

The frozen tissue sections were removed from the −80 °C ultralow temperature refrigerator and quickly placed in a vacuum dryer for approximately 30 minutes at room temperature. Spatial metabolite resolution was performed via the air-flow-assisted desorption electrospray ionization-mass spectrometric imaging (AFADESI-MSI) platform (Beijing Victor Technology Co., Ltd., Beijing, China) and a Q-Orbitrap mass spectrometer (Q Exactive, Thermo Scientific, U.S.A.) with progressive scanning to obtain distribution profiles in tissue sections. The spray solvent was acetonitrile (MS grade, Thermo Fisher, USA):water (Watson's distilled water (Watson's Group)) = 8:2 (v/v) in negative mode with a flow rate of 5 μL/min. The gas flow rate of the delivery gas was 45 L/min, the spraying voltage was 7 kV, the distance between the surface of the sample and the nebulizer was 3 mm, and the distance between the nebulizer and the ion delivery tube was 3 mm. The mass resolution was 60,000, the mass range was 70–1000 Da, and the capillary temperature was 350 °C. The platform parameters were set as follows: Vx was 0.2 mm/s, Dy was 0.1 mm, and Dt was 7 s. The MSI experiment was carried out with a constant rate of 0.2 mm/s continuously scanning the surface of the section in the x direction and a 10 μm vertical step in the y direction. Data acquisition was performed via the Xcalibur Data Acquisition and Processing System, which sets the data acquisition sequence on the basis of sample size, step spacing, and scanning speed. This information was converted by mass spectrometry image analysis software to obtain a two-dimensional spatial intensity distribution map of ions in liver tissue sections. Ions detected by AFADESI-MSI were annotated via the pySM annotation framework and the in-house SmetDB database (Lumingbio, Shanghai, China).

#### Local metabolite extraction.

The raw files collected from the samples in the 6w and 12w groups were converted into imzML format, and ion image reconstruction after background subtraction was performed via the Cardinal software package. All mass spectrometry images were normalized for each pixel via total ion count normalization. Through MSireader software, the generated data were matched with high spatial resolution H&E images, and after accurately extracting the coordinate information of the egg granuloma tissue region as well as the surrounding normal tissue region in each sample, the metabolite information at the pixel points within the region was extracted for comparative analysis.

### Data processing and statistical analysis

#### Intergroup analysis.

The experimental data are presented as the means ± SDs. SPSS 22.0 software was used to analyze the data statistically, and the results were analyzed by independent samples t tests for comparisons between two groups. One-way ANOVA and Dunnett’s test were used to compare the significance of differences in the data among multiple groups, with a significance level of α = 0.05, and *P* < 0.05 was considered statistically significant for the differences in the results of these experiments. (^*^P < 0.05, ^**^P < 0.01, ^***^P < 0.001, ^****^P < 0.0001), and GraphPad Prism 9.0 was used to visualize and analyze the data.

#### Multivariate statistical analysis.

The multivariate statistical analysis began with unsupervised principal component analysis (PCA) to assess both inter- and intragroup variability within the samples. This allowed for the observation of trends in the overall distribution among the samples, identification of potential points of dispersion, and assessment of the stability of the analysis process. A supervised orthogonal partial least squares analysis (OPLS-DA) was then conducted to differentiate overall differences in metabolic profiles between groups. In OPLS-DA, the overall contribution of each variable to group differentiation is ranked by calculating the variable importance of projection (VIP). The VIP score reflects the importance of the first two principal components of the OPLS-DA model in classifying the samples. A VIP value greater than 1 indicates that the variable has a significant effect in discriminating between groups. Student's t test was further used to verify whether the differentially abundant metabolites between groups were significant. Variables satisfying both VIP > 1 and *P* < 0.05 were considered potentially differentially abundant metabolites.

#### Metabolic pathway analysis.

The KEGG IDs of the differentially abundant metabolites were identified for enrichment analysis of metabolic pathways to obtain the enrichment results of metabolic pathways, and the metabolic pathways with *P* < 0.05 were screened as the significantly enriched pathways of the differentially abundant metabolites via a hypergeometric test. The smaller the *P* value is, the more significant the variability in metabolic pathways.

## Supporting information

S1 Fig Visualization of a Metabolomics Dataset in 6w vs control or 12w vs control comparisons. (A) The PCA score plot in 6w vs control comparisons. (B) The OPLS‐DA score plot in 6w vs control comparisons. (C) The volcano plot in 6w vs control comparisons. (D) The PCA score plot in 12w vs control comparisons. (E) The OPLS‐DA score plot in 12w vs control comparisons. (F) The volcano plot showing differential metabolites in the granulomatous tissue and unaffected tissue in 6w mice infected with *S. japonicum*. (G) Bubble diagram of the top 16 ranked metabolism pathway from the comparison between the 6w group and the control group. (H) Bubble diagram of the top 20 ranked metabolism pathway from the comparison between the 12w group and the control group.
(TIF)

S2 Fig Visualization of a Metabolomics Dataset in 6w vs control or 12w vs control comparisons in the granulomatous tissue and unaffected tissue in 6w mice and 12w mice infected with *S. japonicum.
* (A) Representative mass spectra of liver tissue in the granulomatous tissue and unaffected tissue in 6w mice infected with *S. japonicum*. (B) The PCA score plot in the granulomatous tissue and unaffected tissue in 6w mice infected with *S. japonicum*. (C) The OPLS‐DA score plot in the granulomatous tissue and unaffected tissue in 6w mice infected with *S. japonicum*. (D) The volcano plot showing differential metabolites in the granulomatous tissue and unaffected tissue in 6w mice infected with *S. japonicum*. (E) Representative mass spectra of liver tissue in the granulomatous tissue and unaffected tissue in 12w mice infected with *S. japonicum*. (F) The PCA score plot in the granulomatous tissue and unaffected tissue in 12w mice infected with *S. japonicum*. (G) The OPLS‐DA score plot in the granulomatous tissue and unaffected tissue in 12w mice infected with *S. japonicum*. (H) The volcano plot showing differential metabolites in the granulomatous tissue and unaffected tissue in 12w mice infected with *S. japonicum*.
(TIF)

S3 Fig In situ visualization of crucial metabolites and metabolism pathway in the liver of 6w group. (A) In situ visualization of up-regulate ions and down-regulate ions in the 6w mice infected with *S. japonicum*. (B) Bubble diagram of the top 20 ranked metabolism pathway from the comparison between the granulomatous tissue and unaffected tissue in 6w mice infected with *S. japonicum*.
(TIF)

S4 Fig In situ visualization of crucial metabolites and metabolism pathway in the liver of 12w group. (A) In situ visualization of up-regulate ions and down-regulate ions in the 12w mice infected with *S. japonicum*. (B) Bubble diagram of the top 20 ranked metabolism pathway from the comparison between the granulomatous tissue and unaffected tissue in 12w mice infected with *S. japonicum*.
(TIF)

S5 Fig Visualization of liver metabolomics Dataset after PZQ treatment. (A) The PCA score plot after PZQ treatment. (B) The OPLS‐DA score plot after PZQ treatment. (C) Correlation analysis of differentially abundant metabolites associated with PZQ treatment.
(TIF)

S6 Fig Upase1 inhibitors (Benzylacyclouridine, BAU) suppress the activation of LX-2 cells *in vitro.
* (A) Relative mRNA expression of Upase1 in LX-2 cells treated by BAU *in vitro*. (B) The concentration of uridine in LX-2 cells treated by BAU *in vitro*. (C–E) Relative mRNA expression of α-SMA, COL1A1 and COL3A1 in LX-2 cells treated by BAU *in vitro*.
(TIF)

S1 Table Sequences of all primers analyzed by real-time PCR. (DOCX)

S2 Table Discriminating metabolites obtained through the air-flow-assisted desorption electrospray ionization-mass spectrometric imaging (AFADESI-MSI) analysis of the 6w and control groups. (DOCX)

S3 Table Discriminating metabolic pathways obtained through the air-flow-assisted desorption electrospray ionization-mass spectrometric imaging (AFADESI-MSI) analysis of the 6w and control groups. (DOCX)

S4 Table Discriminating metabolites obtained through the air-flow-assisted desorption electrospray ionization-mass spectrometric imaging (AFADESI-MSI) analysis of the 12w and control groups. (DOCX)

S5 Table Discriminating metabolic pathways obtained through the air-flow-assisted desorption electrospray ionization-mass spectrometric imaging (AFADESI-MSI) analysis of the 12w and control groups. (DOCX)

S6 Table Discriminating metabolites obtained through the air-flow-assisted desorption electrospray ionization-mass spectrometric imaging (AFADESI-MSI) analysis of the Granulomatous tissue (6w) and Unaffected tissue. (DOCX)

S7 Table Discriminating metabolic pathways obtained through the air-flow-assisted desorption electrospray ionization-mass spectrometric imaging (AFADESI-MSI) analysis of the Granulomatous tissue (6w) and Unaffected tissue. (DOCX)

S8 Table Discriminating metabolites obtained through the air-flow-assisted desorption electrospray ionization-mass spectrometric imaging (AFADESI-MSI) analysis of the Granulomatous tissue (12w) and Unaffected tissue. (DOCX)

S9 Table Discriminating metabolic pathways obtained through the air-flow-assisted desorption electrospray ionization-mass spectrometric imaging (AFADESI-MSI) analysis of the Granulomatous tissue (12w) and Unaffected tissue. (DOCX)

S10 Table Data used for graphing in manuscripts. (XLSX)
