# Supplementary figures and images for: Mass spectrometry imaging reveals spatial metabolic variation and the crucial role of uridine metabolism in liver injury caused by Schistosoma japonicum
Source: PLoS Negl Trop Dis. 2025 Feb 11;19(2):e0012854. doi: 10.1371/journal.pntd.0012854 (PMC11813095; doi:10.1371/journal.pntd.0012854)

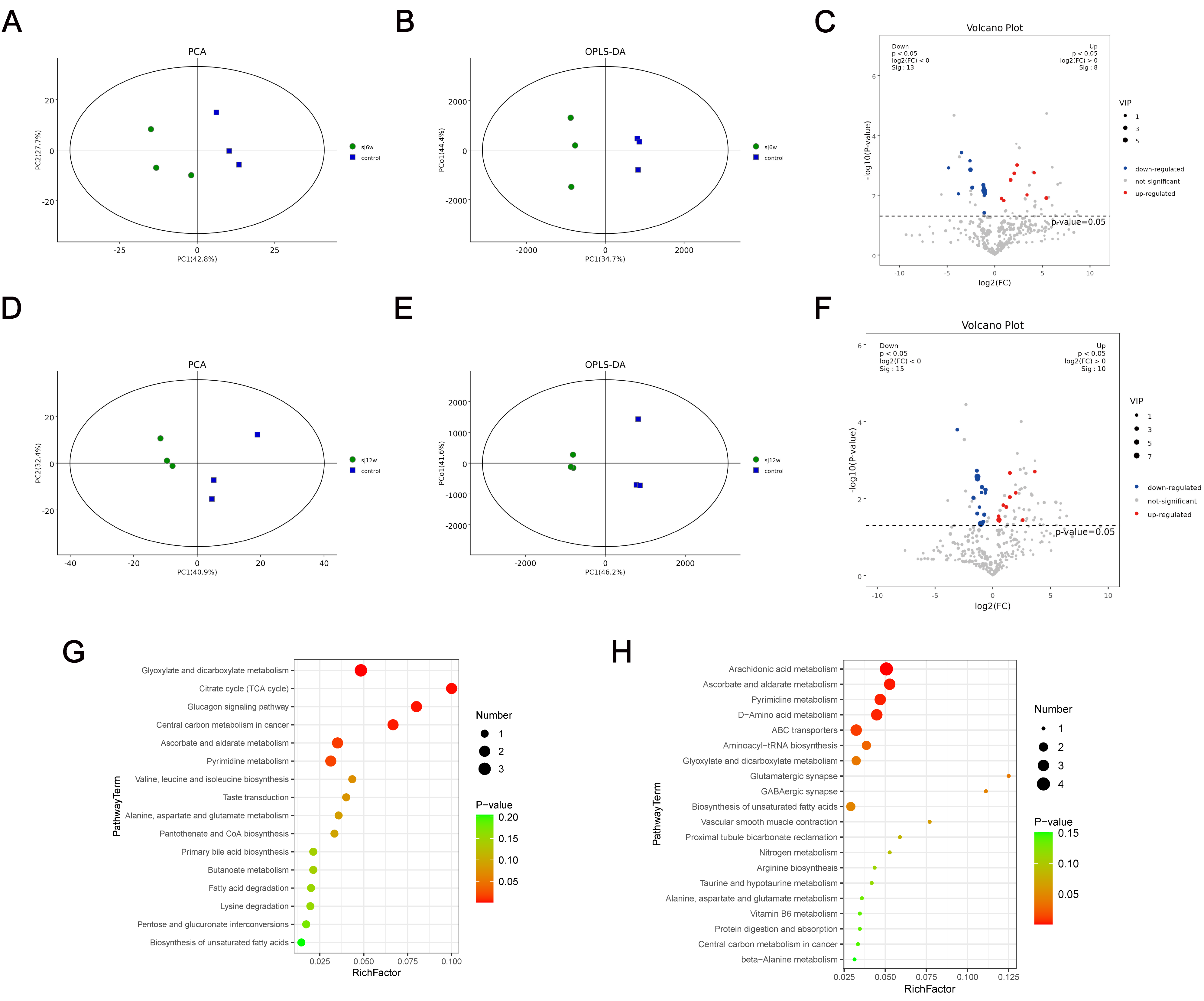

Supplement: S1 Fig — (A) The PCA score plot in 6w vs control comparisons. (B) The OPLS‐DA score plot in 6w vs control comparisons. (C) The volcano plot in 6w vs control comparisons. (D) The PCA score plot in 12w vs control comparisons. (E) The OPLS‐DA score plot in 12w vs control comparisons. (F) The volcano plot showing differential metabolites in the granulomatous tissue and unaffected tissue in 6w mice infected with S. japonicum. (G) Bubble diagram of the top 16 ranked metabolism pathway from the comparison between the 6w group and the control group. (H) Bubble diagram of the top 20 ranked metabolism pathway from the comparison between the 12w group and the control group. (TIF) [file pntd.0012854.s001.tif]

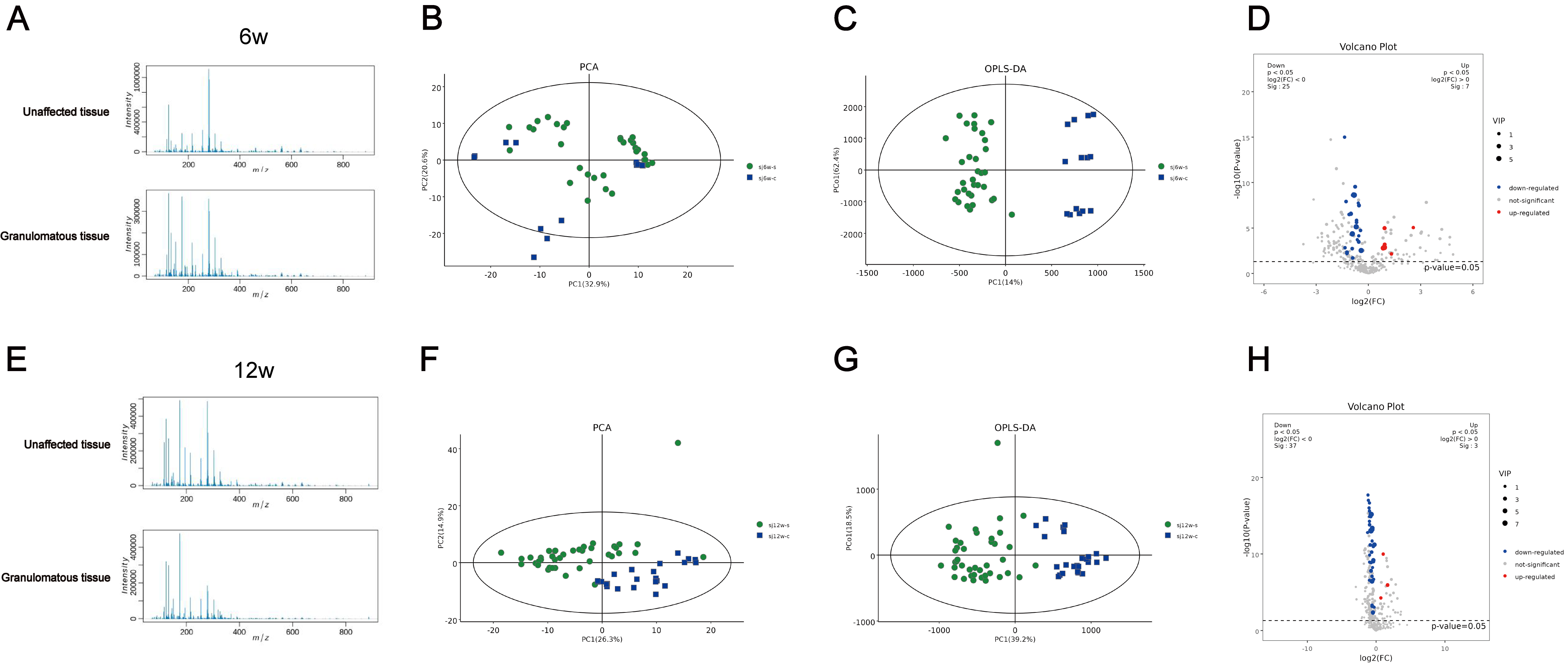

Supplement: S2 Fig — (A) Representative mass spectra of liver tissue in the granulomatous tissue and unaffected tissue in 6w mice infected with S. japonicum. (B) The PCA score plot in the granulomatous tissue and unaffected tissue in 6w mice infected with S. japonicum. (C) The OPLS‐DA score plot in the granulomatous tissue and unaffected tissue in 6w mice infected with S. japonicum. (D) The volcano plot showing differential metabolites in the granulomatous tissue and unaffected tissue in 6w mice infected with S. japonicum. (E) Representative mass spectra of liver tissue in the granulomatous tissue and unaffected tissue in 12w mice infected with S. japonicum. (F) The PCA score plot in the granulomatous tissue and unaffected tissue in 12w mice infected with S. japonicum. (G) The OPLS‐DA score plot in the granulomatous tissue and unaffected tissue in 12w mice infected with S. japonicum. (H) The volcano plot showing differential metabolites in the granulomatous tissue and unaffected tissue in 12w mice infected with S. japonicum. (TIF) [file pntd.0012854.s002.tif]

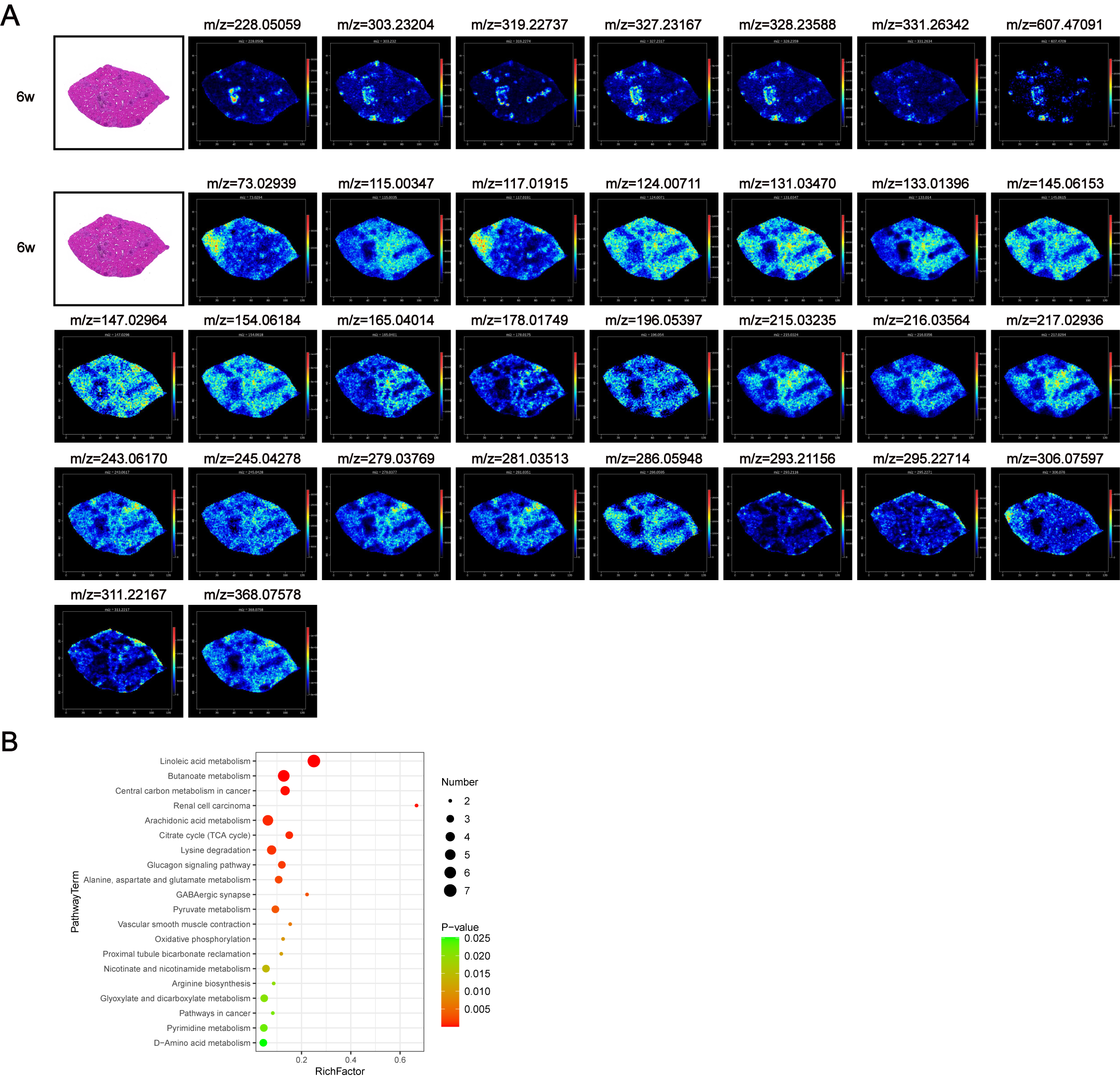

Supplement: S3 Fig — (A) In situ visualization of up-regulate ions and down-regulate ions in the 6w mice infected with S. japonicum. (B) Bubble diagram of the top 20 ranked metabolism pathway from the comparison between the granulomatous tissue and unaffected tissue in 6w mice infected with S. japonicum. (TIF) [file pntd.0012854.s003.tif]

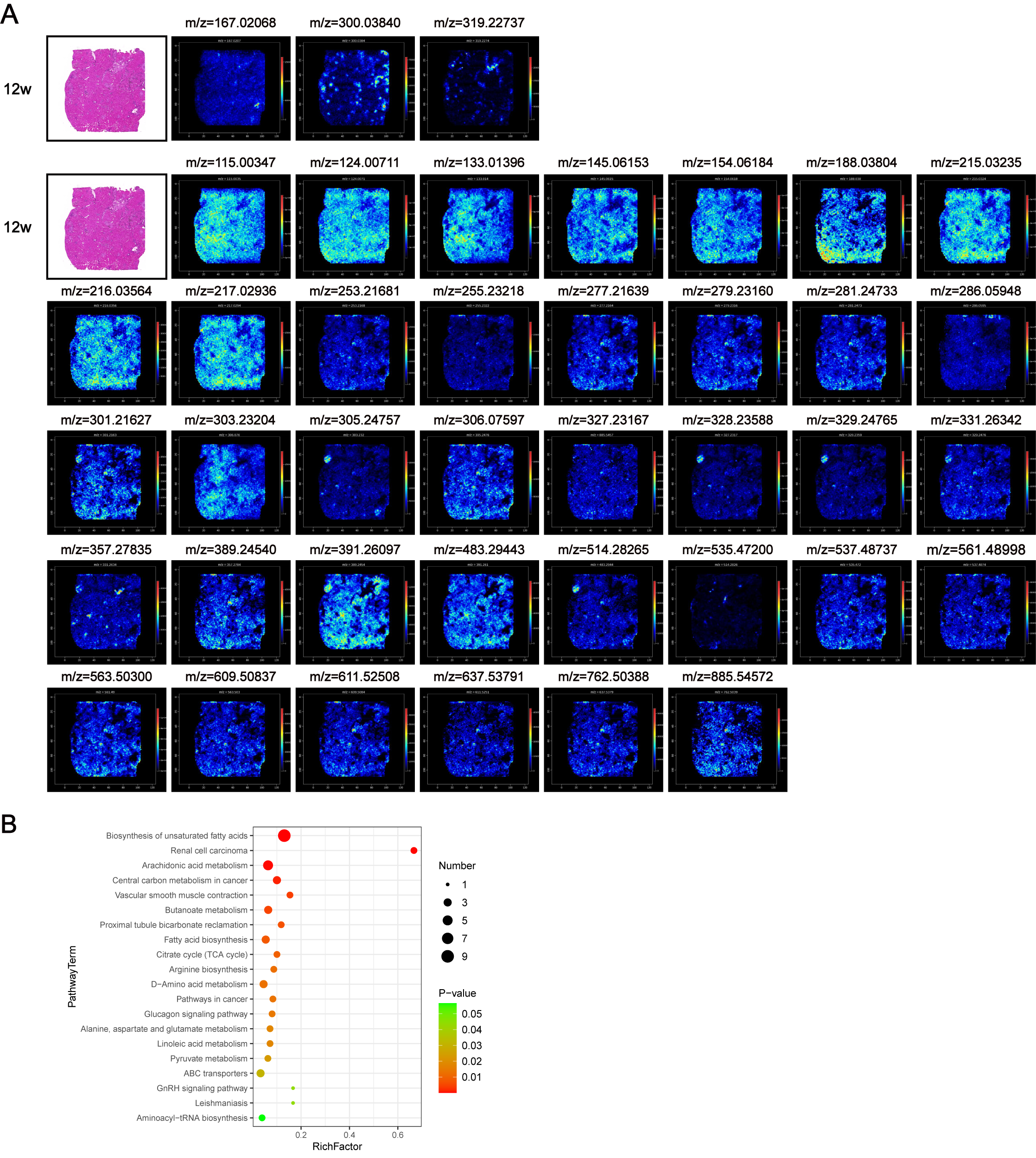

Supplement: S4 Fig — (A) In situ visualization of up-regulate ions and down-regulate ions in the 12w mice infected with S. japonicum. (B) Bubble diagram of the top 20 ranked metabolism pathway from the comparison between the granulomatous tissue and unaffected tissue in 12w mice infected with S. japonicum. (TIF) [file pntd.0012854.s004.tif]

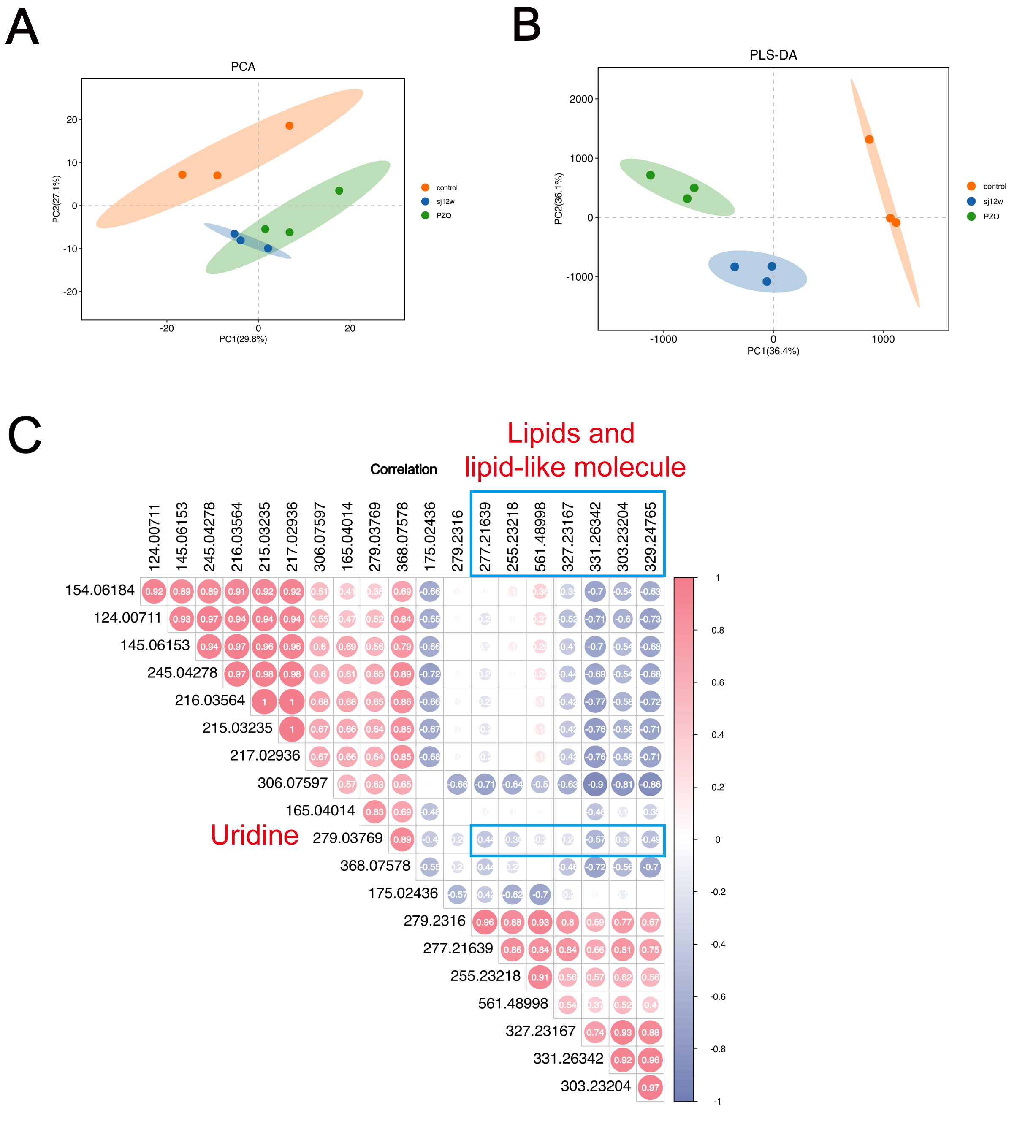

Supplement: S5 Fig — (A) The PCA score plot after PZQ treatment. (B) The OPLS‐DA score plot after PZQ treatment. (C) Correlation analysis of differentially abundant metabolites associated with PZQ treatment. (TIF) [file pntd.0012854.s005.tif]

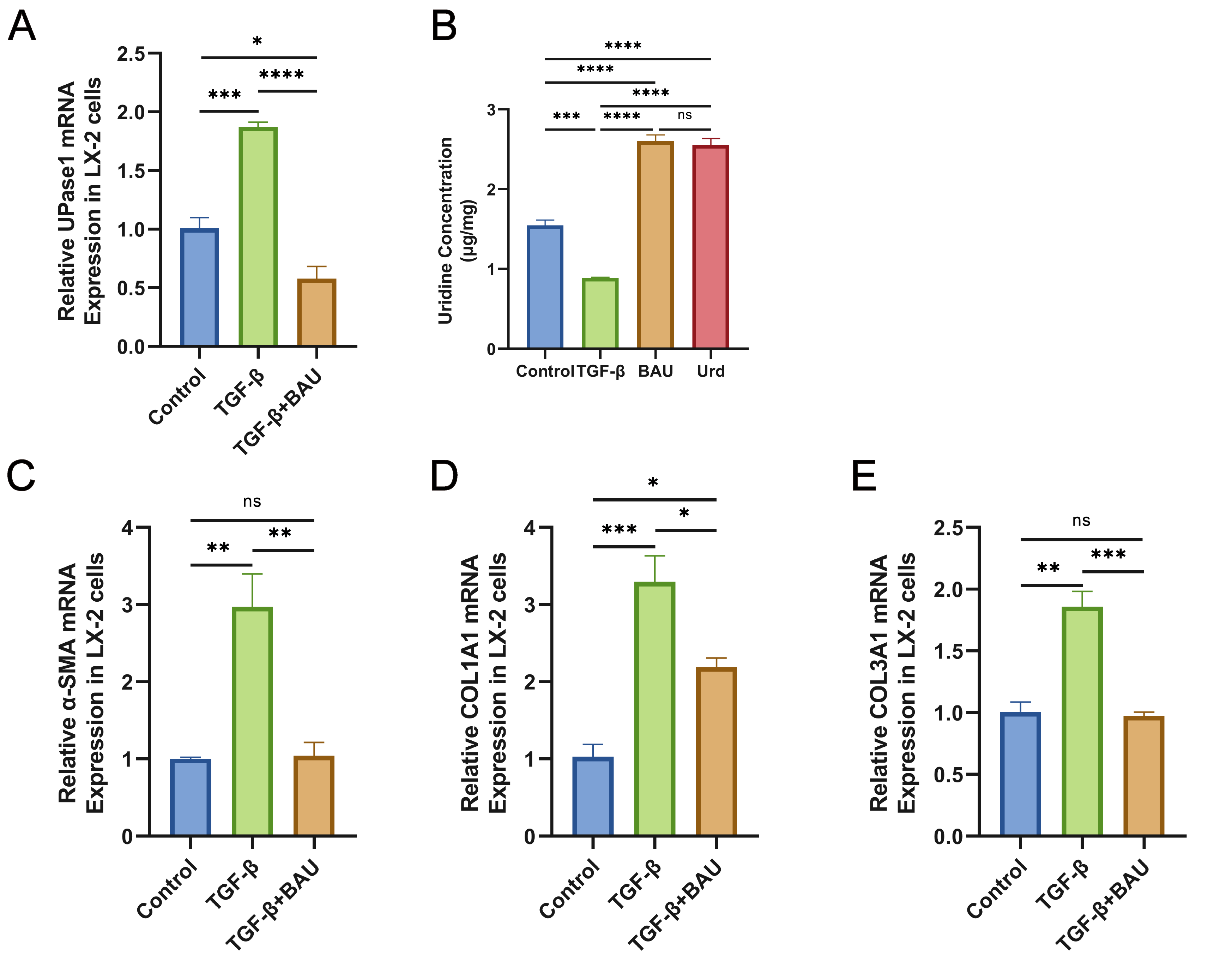

Supplement: S6 Fig — (A) Relative mRNA expression of Upase1 in LX-2 cells treated by BAU in vitro. (B) The concentration of uridine in LX-2 cells treated by BAU in vitro. (C–E) Relative mRNA expression of α-SMA, COL1A1 and COL3A1 in LX-2 cells treated by BAU in vitro. (TIF) [file pntd.0012854.s006.tif]
