# Supplementary material for: Mass spectrometry imaging reveals spatial metabolic variation and the crucial role of uridine metabolism in liver injury caused by Schistosoma japonicum
Source: PLoS Negl Trop Dis. 2025 Feb 11;19(2):e0012854. doi: 10.1371/journal.pntd.0012854 (PMC11813095; doi:10.1371/journal.pntd.0012854)
Supplement: S1 Table — (DOCX) [file pntd.0012854.s007.docx]

| Table S1. Sequences of all primers analyzed by real-time PCR | | | |
| --- | --- | --- | --- |
|  | Gene | Forward (5′ → 3′) | Reverse (5′ → 3′) |
| Human | COL1A1 | AGGGTCACCGTGGCTTCT | TCTGCTGGCTCAGGCTCTT |
|  | COL3A1 | TTGAAGGAGGATGTTCCCATCT | ACAGACACATATTTGGCATGGTT |
|  | α-SMA | CGCATCCTCATCCTCCCT | TGCCAGCAGACTCCATCC |
|  | PPARγ | TGTGAAGGATGCAAGGGTTTCT | ATCCGCCCAAACCTGATGG |
|  | PLIN1 | AATGCCTATGAGAAGGGCGTG | TTCAGCTCAGAAGCAATCTTTTC |
|  | CD36 | CTTTGGCTTAATGAGACTGGGAC | GCAACAAACATCACCACACCA |
|  | SREBP1c | GATCGCGGAGCCATGGATTG | CCAGCATAGGGTGGGTCAAA |
|  | FASN | ACAGCGGGGAATGGGTACT | GACTGGTACAACGAGCGGAT |
|  | Upase1 | ATCTGTGCGGGAACTGACC | TCCAGACCTATCCCACCAGAA |
|  | β-actin | GGCACTCTTCCAGCCTTCC | GAGCCGCCGATCCACAC |
| Mouse | Idh1 | GTGGGCGTCAAGTGTGCTA | CCACCCAGAATGTTTCGGATG |
|  | Idh2 | GGAGAAGCCGGTAGTGGAGAT | GGTCTGGTCACGGTTTGGAA |
|  | Idh3 | TGGGTGTCCAAGGTCTCTC | CTCCCACTGAATAGGTGCTTTG |
|  | Hoga1 | GAGAGTTTCCGTTCCTGACCA | GGCCACGATAGTAACAAGGGG |
|  | Gulo | CAAAACTGGGCGAAGACCTAT | TGCAGGCGATGTCTGAAGG |
|  | Ugdh | TGAAATCAGGGTTACGGTTGTG | TCGACAGGATTCGACTACTTCT |
|  | Upase1 | CCTGGGAAAACGGGTGATTC | CATGGGGAACTCATTCAGGTC |
|  | Upase2 | GAAGGCATGGACGAAGACATT | GCAGACAAACTTTACATCCCCA |
|  | Gls1 | TTCGCCCTCGGAGATCCTAC | CCAAGCTAGGTAACAGACCCT |
|  | Gls2 | TCAGGCATTCCGAAAGAAGTTT | CAGAAGGGGATCTTCGTGTGG |
|  | Cad | CTGCCCGGATTGATTGATGTC | GGTATTAGGCATAGCACAAACCA |
|  | β-actin | CCACCATGTACCCAGGCATT | ACGCAGCTCAGTAACAGTCC |
