# Supplementary material for: Mass spectrometry imaging reveals spatial metabolic variation and the crucial role of uridine metabolism in liver injury caused by Schistosoma japonicum
Source: PLoS Negl Trop Dis. 2025 Feb 11;19(2):e0012854. doi: 10.1371/journal.pntd.0012854 (PMC11813095; doi:10.1371/journal.pntd.0012854)
Supplement: S2 Table — (DOCX) [file pntd.0012854.s008.docx]

**Table S2 Discriminating metabolites obtained through the air-flow-assisted desorption electrospray ionization-mass spectrometric imaging (AFADESI-MSI) analysis of the 6w and control groups.**

| Measured (m/z) | Elemental composition | Adduct^a^ | Delta (ppm) ^b^ | Metabolite identification | AFADESI-MSI | | Fold Change (FC) |
| --- | --- | --- | --- | --- | --- | --- | --- |
|  |  |  |  |  | control | 6w |  |
| 85.02942 | C_3_H_4_ | [M+FA-H]^-^ | 0.96 | Allene | 342.788 | 6026.97 | 17.5822 |
|  |  |  |  | Cyclopropene |  |  |  |
|  |  |  |  | Propyne |  |  |  |
| 129.019 | C_5_H_6_O_4_ | [M-H]^-^ | 1.83 | Glutaconic acid | 2990.29 | 15166 | 5.07175 |
| 131.035 | C_5_H_8_O_4_ | [M-H]^-^ | 2.16 | Methylsuccinic acid | 4369.27 | 17940.4 | 4.10605 |
|  |  |  |  | Monoethyl malonic acid |  |  |  |
|  |  |  |  | Ethylmalonic acid |  |  |  |
|  |  |  |  | Glutaric acid |  |  |  |
|  |  |  |  | 2-Acetolactate |  |  |  |
|  |  |  |  | (S)-2-Acetolactate |  |  |  |
| 142.998 | C_5_H_6_O_6_ | [M-H_2_O-H]^-^ | 1.69 | 4-Hydroxy-2-oxoglutaric acid | 16433.9 | 26874.3 | 1.63529 |
| 173.009 | C_6_H_8_O_7_ | [M-H_2_O-H]^-^ | 1.13 | Citric acid | 8453.98 | 16380.7 | 1.93763 |
|  |  |  |  | Isocitric acid |  |  |  |
|  |  |  |  | Diketogulonic acid |  |  |  |
|  |  |  |  | 2,3-Diketo-L-gulonate |  |  |  |
|  |  |  |  | D-Glucaro-1,4-lactone |  |  |  |
| 186.045 | C_6_H_14_ClNO | [M+Cl]^-^ | 3.65 | 2-((2-Chloroethyl)ethylamino)ethanol | 617.617 | 6466.1 | 10.4694 |
| 228.051 | C_9_H_11_NO_6_ | [M-H]^-^ | 3.38 | 4,5-seco-dopa | 596.344 | 25528.2 | 42.8078 |
| 331.263 | C_22_H_36_O_2_ | [M-H]^-^ | 2.53 | FA(22:4) | 8443.66 | 26647.5 | 3.15591 |
|  |  |  |  | 1-Hydroxy-1-phenyl-3-hexadecanone |  |  |  |
|  |  |  |  | 3-Hydroxy-1-phenyl-1-hexadecanone |  |  |  |
|  |  |  |  | Ethyl Arachidonate |  |  |  |

**Table S2| Continued**

| Measured (m/z) | Elemental composition | Adduct | Delta (ppm) | Metabolite identification | AFADESI-MSI | | Fold Change (FC) |
| --- | --- | --- | --- | --- | --- | --- | --- |
|  |  |  |  |  | control | 6w |  |
| 124.991 | CH_4_O_2_S | [M+FA-H]^-^ | 1.99 | Methanesulfinic acid | 9924.43 | 877.89 | 0.08846 |
| 165.04 | C_5_H_10_O_6_ | [M-H]^-^ | 1.92 | Arabinonic acid | 28062.3 | 12994.1 | 0.46304 |
|  |  |  |  | Ribonic acid |  |  |  |
| 215.032 | C_10_H_9_NaO_4_ | [M-H]^-^ | 1.05 | Sodium ferulate | 404823 | 185036 | 0.45708 |
| 216.036 | C_10_H_13_Cl_2_N | [M-H]^-^ | 1.88 | N,N-Bis(2-chloroethyl)aniline | 25598.2 | 11502 | 0.44933 |
| 217.029 | C_15_H_8_O_3_ | [M-H_2_O-H]^-^ | 1.86 | Coumestan | 129180 | 59021.6 | 0.45689 |
| 245.043 | C_6_H_15_O_8_P | [M-H]^-^ | 1.63 | Glycerophosphoglycerol | 35216.4 | 6803.56 | 0.19319 |
| 254.981 | C_6_H_10_O_10_S | [M-H_2_O-H]^-^ | 0.47 | Glucuronic acid sulfate | 7166.87 | 1179.32 | 0.16455 |
|  |  |  |  | Iduronate 2-sulfate |  |  |  |
|  |  |  |  | (2S,3S,4S,5R)-3,4,5-Trihydroxy-6-sulfooxyoxane-2-carboxylic acid |  |  |  |
| 279.038 | C_9_H_12_N_2_O_6_ | [M+Cl]^-^ | 4.49 | Uridine | 70915.2 | 34604.3 | 0.48797 |
|  |  |  |  | Pseudouridine |  |  |  |
| 281.035 | C_10_H_12_N_4_O_5_S | [M-H_2_O-H]^-^ | 2.44 | Tazobactam | 22909.1 | 10812.1 | 0.47195 |
| 306.076 | C_13_H_13_N_5_O_2_ | [M+Cl]^-^ | 1.17 | zaprinast | 69286.8 | 11837 | 0.17084 |
| 307.079 | C_14_H_17_ClN_4_S | [M-H]^-^ | 0.15 | Clobenpropit | 5897.63 | 417.145 | 0.07073 |
| 368.076 | C_23_H_17_NO_3_S | [M-H_2_O-H]^-^ | 3.41 | 4-[4-(Quinolin-2-ylmethoxy)phenyl]sulfanylbenzoic Acid | 45460.4 | 19711.6 | 0.4336 |
| 498.289 | C_26_H_45_NO_6_S | [M-H]^-^ | 1.67 | Tauroursodeoxycholic acid | 5234.61 | 181.568 | 0.03469 |
|  |  |  |  | Taurodeoxycholic acid |  |  |  |
|  |  |  |  | Taurochenodesoxycholic acid |  |  |  |

^a^ Adduct: The product of two or more different molecules, obtained by addition to each other; ^b^ Delta (ppm): Mass error, (Measured m/z - Theoretical m/z) / m×106, smaller is generally considered more accurate.
