# Supplementary material for: Mass spectrometry imaging reveals spatial metabolic variation and the crucial role of uridine metabolism in liver injury caused by Schistosoma japonicum
Source: PLoS Negl Trop Dis. 2025 Feb 11;19(2):e0012854. doi: 10.1371/journal.pntd.0012854 (PMC11813095; doi:10.1371/journal.pntd.0012854)
Supplement: S3 Table — (DOCX) [file pntd.0012854.s009.docx]

**Table S3 Discriminating metabolic pathways obtained through the air-flow-assisted desorption electrospray ionization-mass spectrometric imaging (AFADESI-MSI) analysis of the 6w and control groups.**

| Pathway name | Related metabolites | Class Ⅱ | Class Ⅰ | *p* | -log(*p*) |
| --- | --- | --- | --- | --- | --- |
| Glyoxylate and dicarboxylate metabolism | Citric acid; 4-Hydroxy-2-oxoglutaric acid; Isocitric acid | Carbohydrate metabolism | Metabolism | 0.0009 | 3.04527 |
| Citrate cycle (TCA cycle) | Citric acid; Isocitric acid | Carbohydrate metabolism | Metabolism | 0.00181 | 2.7423 |
| Glucagon signaling pathway | Citric acid; Isocitric acid | Endocrine system | Organismal Systems | 0.00283 | 2.54784 |
| Central carbon metabolism in cancer | Citric acid; Isocitric acid | Cancer: overview | Human Diseases | 0.00407 | 2.39037 |
| Ascorbate and aldarate metabolism | Arabinonic acid; Diketogulonic acid; 2,3-Diketo-L-gulonate | Carbohydrate metabolism | Metabolism | 0.01423 | 1.84689 |
| Pyrimidine metabolism | Uridine; Pseudouridine | Nucleotide metabolism | Metabolism | 0.01775 | 1.7509 |
