# Supplementary material for: Mass spectrometry imaging reveals spatial metabolic variation and the crucial role of uridine metabolism in liver injury caused by Schistosoma japonicum
Source: PLoS Negl Trop Dis. 2025 Feb 11;19(2):e0012854. doi: 10.1371/journal.pntd.0012854 (PMC11813095; doi:10.1371/journal.pntd.0012854)
Supplement: S4 Table — (DOCX) [file pntd.0012854.s010.docx]

**Table S4 Discriminating metabolites obtained through the air-flow-assisted desorption electrospray ionization-mass spectrometric imaging (AFADESI-MSI) analysis of the 12w and control groups.**

| Measured (m/z) | Elemental composition | Adduct | Delta (ppm) | Metabolite identification | AFADESI-MSI | | Fold Change (FC) |
| --- | --- | --- | --- | --- | --- | --- | --- |
|  |  |  |  |  | control | 12w |  |
| 124.00711 | C_2_H_7_NO_3_S | [M-H]^-^ | 2.21 | Taurine | 725802 | 356376 | 0.49101 |
| 124.99115 | CH_4_O_2_S | [M+FA-H]^-^ | 1.99 | Methanesulfinic acid | 9924.43 | 1172.98 | 0.11819 |
| 145.06153 | C_5_H_10_N_2_O_3_ | [M-H]^-^ | 2.35 | Alanylglycine | 67401.8 | 35202.5 | 0.52228 |
|  |  |  |  | L-Glutamine |  |  |  |
|  |  |  |  | D-Glutamine |  |  |  |
| 154.06184 | C_6_H_9_N_3_O_2_ | [M-H]^-^ | 2.31 | L-Histidine | 49883.3 | 28128.3 | 0.56388 |
| 165.04014 | C_5_H_10_O_6_ | [M-H]^-^ | 1.92 | Arabinonic acid | 28062.3 | 11008.1 | 0.39227 |
|  |  |  |  | Ribonic acid |  |  |  |
| 166.01743 | C_7_H_5_N_3_ | [M+Cl]^-^ | 1.92 | 1,2,3-Benzotriazine | 9621.49 | 4325.77 | 0.44959 |
|  |  |  |  | pyridopyrimidine |  |  |  |
|  |  |  |  | pyrrolodiazepine |  |  |  |
| 215.03235 | C_10_H_9_NaO_4_ | [M-H]^-^ | 1.05 | Sodium ferulate | 404823 | 161211 | 0.39823 |
| 216.03564 | C_10_H_13_C_l2_N | [M-H]^-^ | 1.88 | N,N-Bis(2-chloroethyl)aniline | 25598.2 | 9832.24 | 0.3841 |
| 217.02936 | C_15_H_8_O_3_ | [M-H_2_O-H]^-^ | 1.86 | Coumestan | 129180 | 51588.3 | 0.39935 |
| 245.04278 | C_6_H_15_O_8_P | [M-H]^-^ | 1.63 | Glycerophosphoglycerol | 35216.4 | 11027.2 | 0.31313 |
| 279.03769 | C_9_H_12_N_2_O_6_ | [M+Cl]^-^ | 4.49 | Uridine | 70915.2 | 45738.2 | 0.64497 |
|  |  |  |  | Pseudouridine |  |  |  |
| 281.03513 | C_10_H_12_N_4_O_5_S | [M-H_2_O-H]^-^ | 2.44 | Tazobactam | 22909.1 | 14631.1 | 0.63866 |
| 306.07597 | C_13_H_13_N_5_O_2_ | [M+Cl]^-^ | 1.17 | zaprinast | 69286.8 | 41581.4 | 0.60013 |
| 368.07578 | C_23_H_17_NO_3_S | [M-H_2_O-H]^-^ | 3.41 | 4-[4-(Quinolin-2-ylmethoxy)phenyl]sulfanylbenzoic Acid | 45460.4 | 23549.6 | 0.51803 |
| 483.29443 | C_26_H_44_O_8_ | [M-H]^-^ | 3.96 | Goshonoside F1 | 9481.04 | 4770.74 | 0.50319 |

**Table S4| Continued**

| Measured (m/z) | Elemental composition | Adduct | Delta (ppm) | Metabolite identification | AFADESI-MSI | | Fold Change (FC) |
| --- | --- | --- | --- | --- | --- | --- | --- |
|  |  |  |  |  | control | 12w |  |
|  |  |  |  | Goshonoside F2 |  |  |  |
| 142.99829 | C_5_H_6_O_6_ | [M-H_2_O-H]^-^ | 1.69 | 4-Hydroxy-2-oxoglutaric acid | 16433.9 | 23385.7 | 1.42302 |
| 175.02436 | C_6_H_8_O_6_ | [M-H]^-^ | 2.59 | Ascorbic acid | 295380 | 428160 | 1.44952 |
|  |  |  |  | D-Glucurono-6,3-lactone |  |  |  |
|  |  |  |  | 1,2,3-Propanetricarboxylic acid |  |  |  |
| 228.05059 | C_9_H_11_NO_6_ | [M-H]^-^ | 3.38 | 4,5-seco-dopa | 596.344 | 7457.07 | 12.5046 |
| 300.03840 | C_11_H_11_N_3_O_5_ | [M+Cl]^-^ | 2.90 | Isoniazid alpha-ketoglutaric acid | 2707.43 | 10739 | 3.96648 |
| 319.22737 | C_20_H_32_O_3_ | [M-H]^-^ | 1.56 | 15-HETE | 2527.31 | 15238.8 | 6.02967 |
|  |  |  |  | 16(R)-HETE |  |  |  |
|  |  |  |  | 20-Hydroxyeicosatetraenoic acid |  |  |  |
|  |  |  |  | 18-Hydroxyarachidonic acid |  |  |  |
|  |  |  |  | 19(S)-HETE |  |  |  |
|  |  |  |  | 13-HETE |  |  |  |
|  |  |  |  | 17-HETE |  |  |  |
|  |  |  |  | 12 Hydroxy arachidonic acid |  |  |  |
|  |  |  |  | 15R-hydroxy-5Z,8Z,11Z,13E-eicosatetraenoic acid |  |  |  |
|  |  |  |  | 18-Hydroxy-5Z,8Z,11Z,14Z-eicosatetraenoic acid |  |  |  |
| 329.24765 | C_22_H_34_O_2_ | [M-H]^-^ | 2.91 | FA (22:5) | 12027.5 | 27128.7 | 2.25556 |
|  |  |  |  | 4,8,12,15,19-Docosapentaenoic acid |  |  |  |
| 331.26342 | C_22_H_36_O_2_ | [M-H]^-^ | 2.53 | FA (22:4) | 8443.66 | 23474.9 | 2.78018 |
|  |  |  |  | 1-Hydroxy-1-phenyl-3-hexadecanone |  |  |  |

**Table S4| Continued**

| Measured (m/z) | Elemental composition | Adduct | Delta (ppm) | Metabolite identification | AFADESI-MSI | | Fold Change (FC) |
| --- | --- | --- | --- | --- | --- | --- | --- |
|  |  |  |  |  | control | 12w |  |
|  |  |  |  | 3-Hydroxy-1-phenyl-1-hexadecanone |  |  |  |
| 333.09268 | C_16_H_18_N_2_O_4_S | [M-H]^-^ | 3.68 | Penicillin G | 12434.4 | 18455.4 | 1.48421 |
| 389.24540 | C_21_H_38_O_4_ | [M+Cl]^-^ | 2.59 | MG (18:2) | 8619.01 | 23943 | 2.77793 |
| 391.26097 | C_22_H_36_N_2_O_4_ | [M-H]^-^ | 1.89 | Arterolane | 7925.1 | 14856.1 | 1.87456 |
