# Supplementary material for: Mass spectrometry imaging reveals spatial metabolic variation and the crucial role of uridine metabolism in liver injury caused by Schistosoma japonicum
Source: PLoS Negl Trop Dis. 2025 Feb 11;19(2):e0012854. doi: 10.1371/journal.pntd.0012854 (PMC11813095; doi:10.1371/journal.pntd.0012854)
Supplement: S5 Table — (DOCX) [file pntd.0012854.s011.docx]

**Table S5 Discriminating metabolic pathways obtained through the air-flow-assisted desorption electrospray ionization-mass spectrometric imaging (AFADESI-MSI) analysis of the 12w and control groups.**

| Pathway name | Related metabolites | Class Ⅱ | Class Ⅰ | *p* | -log(*p*) |
| --- | --- | --- | --- | --- | --- |
| Arachidonic acid metabolism | 15-HETE; 16(R)-HETE; 20-Hydroxyeicosatetraenoic acid; 19(S)-HETE | Lipid metabolism | Metabolism | 0.00054 | 3.2647 |
| Ascorbate and aldarate metabolism | Ascorbic acid; D-Glucurono-6,3-lactone; Arabinonic acid | Carbohydrate metabolism | Metabolism | 0.0027 | 2.56934 |
| Pyrimidine metabolism | Uridine; Pseudouridine; L-Glutamine | Nucleotide metabolism | Metabolism | 0.00375 | 2.42544 |
| D-Amino acid metabolism | L-Histidine; L-Glutamine; D-Glutamine | Metabolism of other amino acids | Metabolism | 0.00428 | 2.36892 |
| ABC transporters | Taurine; L-Glutamine | Membrane transport | Environmental Information Processing | 0.01067 | 1.97173 |
| Aminoacyl-tRNA biosynthesis | L-Glutamine; L-Histidine | Translation | Genetic Information Processing | 0.02779 | 1.55611 |
| Glyoxylate and dicarboxylate metabolism | 4-Hydroxy-2-oxoglutaric acid; L-Glutamine | Carbohydrate metabolism | Metabolism | 0.03847 | 1.41486 |
| Glutamatergic synapse | L-Glutamine | Nervous system | Organismal Systems | 0.03999 | 1.398 |
| GABAergic synapse | L-Glutamine | Nervous system | Organismal Systems | 0.04489 | 1.34788 |
| Biosynthesis of unsaturated fatty acids | FA (22:4); FA (22:5) | Lipid metabolism | Metabolism | 0.04675 | 1.33023 |
