# Supplementary material for: Mass spectrometry imaging reveals spatial metabolic variation and the crucial role of uridine metabolism in liver injury caused by Schistosoma japonicum
Source: PLoS Negl Trop Dis. 2025 Feb 11;19(2):e0012854. doi: 10.1371/journal.pntd.0012854 (PMC11813095; doi:10.1371/journal.pntd.0012854)
Supplement: S6 Table — (DOCX) [file pntd.0012854.s012.docx]

**Table S6 Discriminating metabolites obtained through the air-flow-assisted desorption electrospray ionization-mass spectrometric imaging (AFADESI-MSI) analysis of the Granulomatous tissue (6w) and Unaffected tissue.**

| Measured (m/z) | Elemental composition | Adduct | Delta (ppm) | Metabolite identification | AFADESI-MSI | | Fold Change (FC) |
| --- | --- | --- | --- | --- | --- | --- | --- |
|  |  |  |  |  | Unaffected tissue | Granulomatous tissue (6w) |  |
| 228.05059 | C_9_H_11_NO_6_ | [M-H]^-^ | 3.38294 | 4,5-seco-dopa | 15328.6 | 29319.9 | 1.91276 |
| 303.23204 | C_20_H_32_O_2_ | [M-H]^-^ | 3.00983 | FA (20:4)  Cis-8,11,14,17-Eicosatetraenoic acid  Mesterolone  Copalic acid  7,13-Eperudien-15-oic acid | 155898 | 293592 | 1.88323 |
| 319.22737 | C_20_H_32_O_3_ | [M-H]^-^ | 1.56437 | 15-HETE  16(R)-HETE  20-Hydroxyeicosatetraenoic acid  18-Hydroxyarachidonic acid  19(S)-HETE  13-HETE  17-HETE  12 Hydroxy arachidonic acid  15R-hydroxy-5Z,8Z,11Z,13E-eicosatetraenoic acid  18-Hydroxy-5Z,8Z,11Z,14Z-eicosatetraenoic acid | 5092.44 | 12742.2 | 2.50219 |
| 327.23167 | C_22_H_32_O_2_ | [M-H]^-^ | 3.93272 | FA (22:6)  Neogrifolin  Grifolin | 90927.2 | 163508 | 1.79823 |

**Table S6| Continued**

| Measured (m/z) | Elemental composition | Adduct | Delta (ppm) | Metabolite identification | AFADESI-MSI | | Fold Change (FC) |
| --- | --- | --- | --- | --- | --- | --- | --- |
|  |  |  |  |  | Unaffected tissue | Granulomatous tissue (6w) |  |
|  |  |  |  | Retinol acetate |  |  |  |
| 328.23588 | C_15_H_33_N_5_O_4_ | [M-H2O-H]^-^ | 3.09418 | Carrageenan, potassium salt of | 21360.9 | 38426.2 | 1.7989 |
| 331.26342 | C_22_H_36_O_2_ | [M-H]^-^ | 2.52712 | FA (22:4)  1-Hydroxy-1-phenyl-3-hexadecanone  3-Hydroxy-1-phenyl-1-hexadecanone  Ethyl Arachidonate | 19725.2 | 37537.7 | 1.90303 |
| 607.47091 | C_40_H_66_O_5_ | [M-H2O-H]^-^ | 2.83925 | DG (37:6)  DG (36:6) | 729.172 | 4359.38 | 5.97853 |
| 73.02939 | C_2_H_4_ | [M+FA-H]^-^ | 1.52643 | Ethylene | 14835.8 | 9263.69 | 0.62441 |
| 115.00347 | C_4_H_4_O_4_ | [M-H]^-^ | 1.89082 | Fumaric acid  Maleic acid | 29332 | 21459.5 | 0.73161 |
| 117.01915 | C_4_H_6_O_4_ | [M-H]^-^ | 1.59449 | Succinic acid | 187464 | 115441 | 0.61581 |
| 124.00711 | C_2_H_7_NO_3_S | [M-H]^-^ | 2.20919 | Taurine | 487625 | 365730 | 0.75002 |
| 131.03470 | C_5_H_8_O_4_ | [M-H]^-^ | 2.15913 | Methylsuccinic acid | 22267 | 13138.5 | 0.59005 |
|  |  |  |  | Monoethyl malonic acid |  |  |  |
|  |  |  |  | Ethylmalonic acid |  |  |  |
|  |  |  |  | Glutaric acid  2-Acetolactate |  |  |  |
|  |  |  |  | (S)-2-Acetolactate |  |  |  |
| 133.01396 | C_4_H_6_O_5_ | [M-H]^-^ | 2.12851 | Malic acid  D-Malic acid | 210716 | 111322 | 0.52831 |
| 145.06153 | C_5_H_10_N_2_O_3_ | [M-H]^-^ | 2.34573 | Alanylglycine | 43117.9 | 29370.9 | 0.68118 |

**Table S6| Continued**

| Measured (m/z) | Elemental composition | Adduct | Delta (ppm) | Metabolite identification | AFADESI-MSI | | Fold Change (FC) |
| --- | --- | --- | --- | --- | --- | --- | --- |
|  |  |  |  |  | Unaffected tissue | Granulomatous tissue (6w) |  |
|  |  |  |  | L-Glutamine  D-Glutamine |  |  |  |
| 147.02964 | C_5_H_8_O_5_ | [M-H]^-^ | 1.76719 | Citramalic acid  3-Hydroxyglutaric acid  D-2-Hydroxyglutaric acid  L-2-Hydroxyglutaric acid  Ribonolactone  D-Xylono-1,5-lactone  2-Hydroxyglutarate  3-methylmalate(2-) | 9926.97 | 6372.57 | 0.64195 |
| 154.06184 | C_6_H_9_N_3_O_2_ | [M-H]^-^ | 2.30847 | L-Histidine | 40626.5 | 27917.3 | 0.68717 |
| 165.04014 | C_5_H_10_O_6_ | [M-H]^-^ | 1.92348 | Arabinonic acid  Ribonic acid | 13978.3 | 9522.87 | 0.68126 |
| 178.01749 | C_8_H_6_ClN_3_ | [M-H]^-^ | 1.43754 | 2-[(3-Chlorophenyl)diazenyl]acetonitrile | 10254.7 | 4045.25 | 0.39448 |
| 196.05397 | C_10_H_11_NO | [M+Cl]^-^ | 2.56743 | Tryptophol  (R)-Boschniakine | 5362.45 | 2077.19 | 0.38736 |
| 215.03235 | C_10_H_9_NaO_4_ | [M-H]^-^ | 1.04898 | Sodium ferulate | 227117 | 128554 | 0.56602 |
| 216.03564 | C_10_H_13_Cl_2_N | [M-H]^-^ | 1.88172 | N,N-Bis(2-chloroethyl)aniline | 14249.2 | 7824.61 | 0.54913 |
| 217.02936 | C_15_H_8_O_3_ | [M-H2O-H]^-^ | 1.86098 | Coumestan | 72237.5 | 40982.3 | 0.56733 |
| 243.06170 | C_9_H_14_N_2_O_7_ | [M-H2O-H]^-^ | 0.02666 | Aspartyl-Glutamate  L-beta-aspartyl-L-glutamic acid  amma-Glutamylaspartic acid | 12139.3 | 7582.18 | 0.6246 |

**Table S6| Continued**

| Measured (m/z) | Elemental composition | Adduct | Delta (ppm) | Metabolite identification | AFADESI-MSI | | Fold Change (FC) |
| --- | --- | --- | --- | --- | --- | --- | --- |
|  |  |  |  |  | Unaffected tissue | Granulomatous tissue (6w) |  |
| 245.04278 | C_6_H_15_O_8_P | [M-H]^-^ | 1.63372 | Glycerophosphoglycerol | 7498.15 | 5010.95 | 0.66829 |
| 279.03769 | C_9_H_12_N_2_O_6_ | [M+Cl]^-^ | 4.49065 | Uridine  Pseudouridine | 42271.4 | 21050.1 | 0.49798 |
| 281.03513 | C_10_H_12_N_4_O_5_S | [M-H2O-H]^-^ | 2.43633 | Tazobactam | 13505.2 | 6295.58 | 0.46616 |
| 286.05948 | C_11_H_13_N_3_O_4_ | [M+Cl]^-^ | 1.85855 | 3,N(4)-Ethenodeoxycytidine | 12055.6 | 4962.22 | 0.41161 |
| 293.21156 | C_18_H_30_O_3_ | [M-H]^-^ | 2.2574 | 9-OxoODE | 22198.9 | 9447.84 | 0.4256 |
|  |  |  |  | 9(10)-EpODE |  |  |  |
|  |  |  |  | 9-HOTE |  |  |  |
|  |  |  |  | (2'E,4'Z,8E)-Colneleic acid |  |  |  |
|  |  |  |  | (9S,10E,12Z,15Z)-9-Hydroxy-10,12,15-octadecatrienoic acid |  |  |  |
|  |  |  |  | Sterebin D |  |  |  |
|  |  |  |  | 3,4-Dimethyl-5-pentyl-2-furanheptanoic acid |  |  |  |
|  |  |  |  | 3,4-Dimethyl-5-propyl-2-furannonanoic acid |  |  |  |
|  |  |  |  | 5-Hexyl-2-furanoctanoic acid  5-pentyl-2-furannonanoic acid |  |  |  |
| 295.22714 | C_18_H_34_O_4_ | [M-H2O-H]^-^ | 0.59428 | 9,10-DHOME  12,13-DHOME  Octadecanedioic acid  Dibutyl decanedioate | 32749.8 | 17558.6 | 0.53614 |

**Table S6| Continued**

| Measured (m/z) | Elemental composition | Adduct | Delta (ppm) | Metabolite identification | AFADESI-MSI | | Fold Change (FC) |
| --- | --- | --- | --- | --- | --- | --- | --- |
|  |  |  |  |  | Unaffected tissue | Granulomatous tissue (6w) |  |
| 306.07597 | C_13_H_13_N_5_O_2_ | [M+Cl]^-^ | 1.16678 | zaprinast | 18718.1 | 9802.42 | 0.52369 |
| 311.22167 | C_18_H_32_O_4_ | [M-H]^-^ | 3.57709 | 11-HpODE | 24234.1 | 10340.6 | 0.42669 |
|  |  |  |  | (10E,12Z)-(9S)-9-Hydroperoxyoctadeca-10,12-dienoic acid |  |  |  |
|  |  |  |  | 13-HPODE(1-) |  |  |  |
|  |  |  |  | 13-L-Hydroperoxylinoleic acid |  |  |  |
|  |  |  |  | 8(R)-Hydroperoxylinoleic acid |  |  |  |
|  |  |  |  | 9(S)-HPODE |  |  |  |
|  |  |  |  | 12,13-DiHODE |  |  |  |
|  |  |  |  | 15,16-DiHODE |  |  |  |
|  |  |  |  | 9,10-DiHODE |  |  |  |
|  |  |  |  | (±)-(E)-13-Hydroxy-10-oxo-11-octadecenoic acid |  |  |  |
| 368.07578 | C_23_H_17_NO_3_S | [M-H2O-H]^-^ | 3.41189 | 4-[4-(Quinolin-2-ylmethoxy)phenyl]sulfanylbenzoic Acid | 23336.8 | 12534.9 | 0.53713 |
