# Supplementary material for: Mass spectrometry imaging reveals spatial metabolic variation and the crucial role of uridine metabolism in liver injury caused by Schistosoma japonicum
Source: PLoS Negl Trop Dis. 2025 Feb 11;19(2):e0012854. doi: 10.1371/journal.pntd.0012854 (PMC11813095; doi:10.1371/journal.pntd.0012854)
Supplement: S7 Table — (DOCX) [file pntd.0012854.s013.docx]

**Table S7 Discriminating metabolic pathways obtained through the air-flow-assisted desorption electrospray ionization-mass spectrometric imaging (AFADESI-MSI) analysis of the Granulomatous tissue (6w) and Unaffected tissue.**

| Pathway name | Related metabolites | Class Ⅱ | Class Ⅰ | *p* | -log(*p*) |
| --- | --- | --- | --- | --- | --- |
| Linoleic acid metabolism | FA (20:4); 11-HpODE; (10E,12Z)-(9S)-9-Hydroperoxyoctadeca-10,12-dienoic acid; 9(S)-HPODE; 13-L-Hydroperoxylinoleic acid; 8(R)-Hydroperoxylinoleic acid; 12,13-DHOME; 9-OxoODE | Lipid metabolism | Metabolism | 3.76425E-09 | 8.424321281 |
| Butanoate metabolism | D-Malic acid; Succinic acid; (S)-2-Acetolactate; Fumaric acid; Maleic acid; 2-Hydroxyglutarate | Carbohydrate metabolism | Metabolism | 3.82162E-06 | 5.417752428 |
| Central carbon metabolism in cancer | Malic acid; Succinic acid; L-Glutamine; Fumaric acid; | Cancer: overview | Human Diseases | 0.000159084 | 3.798372773 |
| Renal cell carcinoma | Malic acid; Fumaric acid | Cancer: specific types | Human Diseases | 0.00026462 | 3.577376536 |
| Arachidonic acid metabolism | FA (20:4); 15-HETE; 16(R)-HETE; 20-Hydroxyeicosatetraenoic acid; 19(S)-HETE | Lipid metabolism | Metabolism | 0.000791314 | 3.101651415 |
| Citrate cycle (TCA cycle) | Malic acid; Succinic acid; Fumaric acid | Carbohydrate metabolism | Metabolism | 0.000812805 | 3.090013415 |
| Lysine degradation | Succinic acid; Glutaric acid; D-2-Hydroxyglutaric acid; L-2-Hydroxyglutaric acid | Amino acid metabolism | Metabolism | 0.001259766 | 2.899709972 |

**Table S7| Continued**

| Pathway name | Related metabolites | Class Ⅱ | Class Ⅰ | *p* | -log(*p*) |
| --- | --- | --- | --- | --- | --- |
| Glucagon signaling pathway | Fumaric acid; Succinic acid; Malic acid | Endocrine system | Organismal Systems | 0.001587388 | 2.799316932 |
| Alanine, aspartate and glutamate metabolism | Succinic acid; L-Glutamine; Fumaric acid | Amino acid metabolism | Metabolism | 0.002217332 | 2.654169285 |
| GABAergic synapse | Succinic acid; L-Glutamine | Nervous system | Organismal Systems | 0.003063324 | 2.513807015 |
| Pyruvate metabolism | Malic acid; Succinic acid; Fumaric acid | Carbohydrate metabolism | Metabolism | 0.003271043 | 2.485313804 |
| Vascular smooth muscle contraction | FA (20:4); 20-Hydroxyeicosatetraenoic acid | Circulatory system | Organismal Systems | 0.006480453 | 2.188394633 |
| Oxidative phosphorylation | Succinic acid; Fumaric acid | Energy metabolism | Metabolism | 0.009793144 | 2.009077838 |
| Proximal tubule bicarbonate reclamation | Malic acid; L-Glutamine | Excretory system | Organismal Systems | 0.011032978 | 1.957307257 |
| Nicotinate and nicotinamide metabolism | Succinic acid; Fumaric acid; Maleic acid | Metabolism of cofactors and vitamins | Metabolism | 0.014186139 | 1.848135793 |
| Arginine biosynthesis | L-Glutamine; Fumaric acid | Amino acid metabolism | Metabolism | 0.019805339 | 1.703217719 |
| Glyoxylate and dicarboxylate metabolism | Malic acid; Succinic acid; L-Glutamine | Carbohydrate metabolism | Metabolism | 0.020542486 | 1.687347003 |
| Pathways in cancer | Malic acid; Fumaric acid | Cancer: overview | Human Diseases | 0.021478005 | 1.668006063 |
| Pyrimidine metabolism | Uridine; Pseudouridine; L-Glutamine | Nucleotide metabolism | Metabolism | 0.022340714 | 1.650902954 |
| D-Amino acid metabolism | L-Histidine; L-Glutamine; D-Glutamine | Metabolism of other amino acids | Metabolism | 0.025195877 | 1.598670528 |

**Table S7| Continued**

| Pathway name | Related metabolites | Class Ⅱ | Class Ⅰ | *p* | -log(*p*) |
| --- | --- | --- | --- | --- | --- |
| Biosynthesis of unsaturated fatty acids | FA (20:4); FA (22:6); FA (22:4) | Lipid metabolism | Metabolism | 0.027204599 | 1.565357664 |
| Tyrosine metabolism | Succinic acid; Fumaric acid; Maleic acid | Amino acid metabolism | Metabolism | 0.03728219 | 1.428498589 |
| Sulfur metabolism | Taurine; Succinic acid | Energy metabolism | Metabolism | 0.0389577 | 1.409406696 |
