# Supplementary material for: Mass spectrometry imaging reveals spatial metabolic variation and the crucial role of uridine metabolism in liver injury caused by Schistosoma japonicum
Source: PLoS Negl Trop Dis. 2025 Feb 11;19(2):e0012854. doi: 10.1371/journal.pntd.0012854 (PMC11813095; doi:10.1371/journal.pntd.0012854)
Supplement: S8 Table — (DOCX) [file pntd.0012854.s014.docx]

**Table S8 Discriminating metabolites obtained through the air-flow-assisted desorption electrospray ionization-mass spectrometric imaging (AFADESI-MSI) analysis of the Granulomatous tissue (12w) and Unaffected tissue.**

| Measured (m/z) | Elemental composition | Adduct | Delta (ppm) | Metabolite identification | AFADESI-MSI | | Fold Change (FC) |
| --- | --- | --- | --- | --- | --- | --- | --- |
|  |  |  |  |  | Unaffected tissue | Granulomatous tissue (12w) |  |
| 167.02068 | C_5_H_4_N_4_O_3_ | [M-H]^-^ | 2.312177 | Uric acid | 9589.159 | 15438.48 | 1.609993 |
| 300.03840 | C_11_H_11_N_3_O_5_ | [M+Cl]^-^ | 2.901398 | Isoniazid alpha-ketoglutaric acid | 7391.571 | 14997.73 | 2.029032 |
| 319.22737 | C_20_H_32_O_3_ | [M-H]^-^ | 1.564365 | 15-HETE  16(R)-HETE  20-Hydroxyeicosatetraenoic acid  18-Hydroxyarachidonic acid  19(S)-HETE  13-HETE  17-HETE  12 Hydroxy arachidonic acid  15R-hydroxy-5Z,8Z,11Z,13E-eicosatetraenoic acid  18-Hydroxy-5Z,8Z,11Z,14Z-eicosatetraenoic acid | 8042.624 | 25488.59 | 3.169189 |
| 115.00347 | C_4_H_4_O_4_ | [M-H]^-^ | 1.890824 | Fumaric acid  Maleic acid | 38421.92 | 29681.89 | 0.772525 |
| 124.00711 | C_2_H_7_NO_3_S | [M-H]^-^ | 2.209191 | Taurine | 393874.7 | 331798.6 | 0.842396 |
| 133.01396 | C_4_H_6_O_5_ | [M-H]^-^ | 2.128508 | Malic acid  D-Malic acid | 308862.7 | 203768 | 0.659736 |
| 145.06153 | C_5_H_10_N_2_O_3_ | [M-H]^-^ | 2.345734 | Alanylglycine | 39956.72 | 30087.71 | 0.753007 |
|  |  |  |  | L-Glutamine |  |  |  |

**Table S8| Continued**.

| Measured (m/z) | Elemental composition | Adduct | Delta (ppm) | Metabolite identification | AFADESI-MSI | | Fold Change (FC) |
| --- | --- | --- | --- | --- | --- | --- | --- |
|  |  |  |  |  | Unaffected tissue | Granulomatous tissue (12w) |  |
|  |  |  |  | D-Glutamine |  |  |  |
| 154.06184 | C_6_H_9_N_3_O_2_ | [M-H]^-^ | 2.308474 | L-Histidine | 34376.31 | 24422.87 | 0.710456 |
| 188.03804 | C_7_H_13_NO_4_S | [M-H2O-H]^-^ | 0.50295 | (2R,2'S)-Isobuteine  2-Methyl-2-[(1-oxo-2-propenyl)amino]-1-propanesulfonic acid | 7187.726 | 3684.313 | 0.512584 |
| 215.03235 | C_10_H_9_NaO_4_ | [M-H]^-^ | 1.048981 | Sodium ferulate | 190811.6 | 144270.7 | 0.75609 |
| 216.03564 | C_10_H_13_Cl_2_N | [M-H]^-^ | 1.881719 | N,N-Bis(2-chloroethyl)aniline | 11885.16 | 8677.621 | 0.730122 |
| 217.02936 | C_15_H_8_O_3_ | [M-H2O-H]^-^ | 1.860977 | Coumestan | 61093.91 | 46320.46 | 0.758185 |
| 253.21681 | C_16_H_30_O_2_ | [M-H]^-^ | 1.964894 | Hypogeic acid  FA (16:1)  Palmitelaidic acid  (E)-6-Hexadecenoic acid  (Z)-5-Hexadecenoic acid  (E)-3-Hexadecenoic acid  (Z)-13-Hexadecenoic acid  (E)-11-Hexadecenoic acid  (Z)-14-Methyl-6-pentadecenoic acid | 32013.71 | 20890.63 | 0.652552 |
| 255.23218 | C_16_H_32_O_2_ | [M-H]^-^ | 3.035158 | FA (16:0) | 204944.5 | 138783 | 0.677173 |
|  |  |  |  | Trimethyltridecanoic acid |  |  |  |
|  |  |  |  | Isopalmitic acid |  |  |  |
|  |  |  |  | Butyl dodecanoate |  |  |  |
|  |  |  |  | Hexyl decanoate |  |  |  |

**Table S8| Continued**

| Measured (m/z) | Elemental composition | Adduct | Delta (ppm) | Metabolite identification | AFADESI-MSI | | Fold Change (FC) |
| --- | --- | --- | --- | --- | --- | --- | --- |
|  |  |  |  |  | Unaffected tissue | Granulomatous tissue (12w) |  |
| 277.21639 | C_18_H_30_O_2_ | [M-H]^-^ | 3.29389 | FA (18:3)  Calendic acid  Punicic acid  Linolenelaidic acid | 35435.55 | 19596.86 | 0.553028 |
| 279.23160 | C_13_H_32_N_4_ | [M+Cl]^-^ | 1.783182 | N1,N11-Bis(ethyl)norspermine | 477746.3 | 282847.2 | 0.592045 |
| 281.24733 | C_18_H_34_O_2_ | [M-H]^-^ | 4.543935 | FA (18:1)  Elaidic acid  Petroselinic acid  (Z)-13-Octadecenoic acid  7Z-octadecenoic acid  Octadec-9-enoic Acid | 340845.1 | 207556.3 | 0.608946 |
| 286.05948 | C_11_H_13_N_3_O_4_ | [M+Cl]^-^ | 1.858547 | 3,N(4)-Ethenodeoxycytidine | 12760.08 | 8505.024 | 0.666534 |
| 301.21627 | C_20_H_30_O_2_ | [M-H]^-^ | 3.417537 | FA (20:5) | 6993.724 | 3574.496 | 0.5111 |
|  |  |  |  | Retinyl ester |  |  |  |
|  |  |  |  | 8,15-Isopimaradien-18-oic acid |  |  |  |
|  |  |  |  | Siderone |  |  |  |
|  |  |  |  | Yucalexin B14 |  |  |  |
|  |  |  |  | Yucalexin A16 |  |  |  |
|  |  |  |  | 8,13-Abietadien-18-oic acid |  |  |  |
|  |  |  |  | ent-8(17),13(16),14-Labdatrien-18-oic acid |  |  |  |
|  |  |  |  | Isopimaric acid |  |  |  |
| 303.23204 | C_20_H_32_O_2_ | [M-H]^-^ | 3.009831 | FA (20:4) | 218435.1 | 158537.1 | 0.725786 |

**Table S8| Continued**

| Measured (m/z) | Elemental composition | Adduct | Delta (ppm) | Metabolite identification | AFADESI-MSI | | Fold Change (FC) |
| --- | --- | --- | --- | --- | --- | --- | --- |
|  |  |  |  |  | Unaffected tissue | Granulomatous tissue (12w) |  |
|  |  |  |  | Cis-8,11,14,17-Eicosatetraenoic acid  Mesterolone  Copalic acid  7,13-Eperudien-15-oic acid |  |  |  |
| 305.24757 | C_20_H_34_O_2_ | [M-H]^-^ | 3.372259 | FA (20:3)  5,8,11-Eicosatrienoic acid  Sciadonic acid | 29662.58 | 19564.39 | 0.659565 |
| 306.07597 | C_13_H_13_N_5_O_2_ | [M+Cl]^-^ | 1.166784 | zaprinast | 56086.26 | 33493.45 | 0.597178 |
| 327.23167 | C_22_H_32_O_2_ | [M-H]^-^ | 3.932715 | FA (22:6)  Neogrifolin  Grifolin  Retinol acetate | 88022.65 | 59659.8 | 0.677778 |
| 328.23588 | C_15_H_33_N_5_O_4_ | [M-H2O-H]^-^ | 3.094183 | Carrageenan, potassium salt of | 20502.29 | 13564.92 | 0.661629 |
| 329.24765 | C_22_H_34_O_2_ | [M-H]^-^ | 2.910254 | 22:5  FA (22:5)  4,8,12,15,19-Docosapentaenoic acid | 34993.32 | 22221.81 | 0.63503 |
| 331.26342 | C_22_H_36_O_2_ | [M-H]^-^ | 2.527117 | FA (22:4)  1-Hydroxy-1-phenyl-3-hexadecanone  3-Hydroxy-1-phenyl-1-hexadecanone  Ethyl Arachidonate | 27328.12 | 22276.65 | 0.815155 |
| 357.27835 | C_24_H_38_O_2_ | [M-H]^-^ | 4.337618 | Tetracosapentaenoic acid (24:5n-6) | 7069.384 | 3641.143 | 0.515058 |
|  |  |  |  | Tetracosapentaenoic acid (24:5n-3) |  |  |  |

**Table S8| Continued**

| Measured (m/z) | Elemental composition | Adduct | Delta (ppm) | Metabolite identification | AFADESI-MSI | | Fold Change (FC) |
| --- | --- | --- | --- | --- | --- | --- | --- |
|  |  |  |  |  | Unaffected tissue | Granulomatous tissue (12w) |  |
| 389.24540 | C_21_H_38_O_4_ | [M+Cl]^-^ | 2.58778 | MG (18:2) | 32358.23 | 19454.17 | 0.601212 |
| 391.26097 | C_22_H_36_N_2_O_4_ | [M-H]^-^ | 1.891304 | Arterolane | 20023.33 | 11644.42 | 0.581543 |
| 483.29443 | C_26_H_44_O_8_ | [M-H]^-^ | 3.959094 | Goshonoside F1  Goshonoside F2 | 6973.041 | 3512.064 | 0.503663 |
| 514.28265 | C_29_H_41_NO_7_ | [M-H]^-^ | 3.147543 | Candoxatril | 17851.7 | 13716.27 | 0.768346 |
| 535.47200 | C_34_H_64_O_4_ | [M-H]^-^ | 2.207426 | FAHFA (18:1(9Z)/6-O-16:0) | 11798.47 | 5999.124 | 0.508466 |
| 537.48737 | C_34_H_66_O_4_ | [M-H]^-^ | 2.726748 | FAHFA (16:0/9-O-18:0) | 9622.97 | 4884.75 | 0.50762 |
|  |  |  |  | FAHFA (16:0/5-O-18:0) |  |  |  |
|  |  |  |  | FAHFA (16:0/10-O-18:0) |  |  |  |
|  |  |  |  | FAHFA (16:0/12-O-18:0) |  |  |  |
|  |  |  |  | FAHFA (16:0/7-O-18:0) |  |  |  |
|  |  |  |  | FAHFA (16:0/8-O-18:0) |  |  |  |
|  |  |  |  | FAHFA (16:0/11-O-18:0) |  |  |  |
|  |  |  |  | FAHFA (16:0/13-O-18:0) |  |  |  |
|  |  |  |  | FAHFA (18:0/5-O-16:0) |  |  |  |
|  |  |  |  | FAHFA (18:0/7-O-16:0) |  |  |  |
|  |  |  |  | FAHFA (18:0/8-O-16:0) |  |  |  |
|  |  |  |  | FAHFA (18:0/9-O-16:0) |  |  |  |
|  |  |  |  | FAHFA (18:0/10-O-16:0) |  |  |  |
|  |  |  |  | FAHFA (18:0/11-O-16:0) |  |  |  |
|  |  |  |  | FAHFA (18:0/12-O-16:0) |  |  |  |
|  |  |  |  | FAHFA (18:0/13-O-16:0) |  |  |  |

**Table S8| Continued**

| Measured (m/z) | Elemental composition | Adduct | Delta (ppm) | Metabolite identification | AFADESI-MSI | | Fold Change (FC) |
| --- | --- | --- | --- | --- | --- | --- | --- |
|  |  |  |  |  | Unaffected tissue | Granulomatous tissue (12w) |  |
|  |  |  |  | FAHFA (16:0/6-O-18:0)  FAHFA (18:0/6-O-16:0) |  |  |  |
| 561.48998 | C_36_H_68_O_5_ | [M-H2O-H]^-^ | 3.02439 | DG (33:1) | 19696 | 10137.92 | 0.51472 |
| 563.50300 | C_36_H_68_O_4_ | [M-H]^-^ | 2.631089 | FAHFA (18:1(9Z)/12-O-18:0) | 10552.66 | 5355.371 | 0.50749 |
| 609.50837 | C_37_H_70_O_6_ | [M-H]^-^ | 2.61163 | Glycerol 1,3-didodecanoate 2-decanoate  Glycerol 1-dodecanoate 2-tetradecanoate 3-octanoate  TG (34:0) | 10250.23 | 5009.046 | 0.488676 |
| 611.52508 | C_36_H_70_O_4_ | [M+FA-H]^-^ | 0.868009 | FAHFA (18:0/9-O-18:0)  FAHFA (18:0/12-O-18:0)  FAHFA (18:0/5-O-18:0)  FAHFA (18:0/7-O-18:0)  FAHFA (18:0/8-O-18:0)  FAHFA (18:0/10-O-18:0)  FAHFA (18:0/11-O-18:0)  FAHFA (18:0/13-O-18:0)  FAHFA (18:0/6-O-18:0) | 5664.91 | 2477.839 | 0.437401 |
| 637.53791 | C_46_H_70_O | [M-H]^-^ | 3.948196 | 2-octaprenylphenol | 7932.864 | 3608.339 | 0.45486 |
| 762.504 | C_39_H_67_N_5_O_7_ | [M+FA-H]^-^ | 2.137597 | Mmae | 5655.636 | 2382.176 | 0.421204 |
| 885.54572 | C_47_H_83_O_13_P | [M-H]^-^ | 4.666322 | PI (38:4) | 12051.73 | 8404.124 | 0.697338 |
