# Supplementary material for: Mass spectrometry imaging reveals spatial metabolic variation and the crucial role of uridine metabolism in liver injury caused by Schistosoma japonicum
Source: PLoS Negl Trop Dis. 2025 Feb 11;19(2):e0012854. doi: 10.1371/journal.pntd.0012854 (PMC11813095; doi:10.1371/journal.pntd.0012854)
Supplement: S9 Table — (DOCX) [file pntd.0012854.s015.docx]

**Table S9 Discriminating metabolic pathways obtained through the air-flow-assisted desorption electrospray ionization-mass spectrometric imaging (AFADESI-MSI) analysis of the Granulomatous tissue (12w) and Unaffected tissue.**

| Pathway name | Related metabolites | Class Ⅱ | Class Ⅰ | *p* | -log(*p*) |
| --- | --- | --- | --- | --- | --- |
| Biosynthesis of unsaturated fatty acids | FA (18:1); FA (16:0); FA (20:4); FA (22:6); FA (18:3); FA (22:5); FA (20:3); FA (22:4); FA (20:5) | Lipid metabolism | Metabolism | 6.25397E-10 | 9.20384 |
| Renal cell carcinoma | Malic acid; Fumaric acid | Cancer: specific types | Human Diseases | 0.000160278 | 3.79513 |
| Arachidonic acid metabolism | FA (20:4); 15-HETE; 16(R)-HETE; 19(S)-HETE; 20-Hydroxyeicosatetraenoic acid | Lipid metabolism | Metabolism | 0.000237652 | 3.62406 |
| Central carbon metabolism in cancer | Malic acid; L-Glutamine; Fumaric acid | Cancer: overview | Human Diseases | 0.001312248 | 2.88198 |
| Vascular smooth muscle contraction | FA (20:4); 20-Hydroxyeicosatetraenoic acid | Circulatory system | Organismal Systems | 0.00398028 | 2.40009 |
| Butanoate metabolism | D-Malic acid; Fumaric acid; Maleic acid | Carbohydrate metabolism | Metabolism | 0.004819329 | 2.31701 |
| Proximal tubule bicarbonate reclamation | Malic acid; L-Glutamine | Excretory system | Organismal Systems | 0.006814047 | 2.16659 |
| Fatty acid biosynthesis | FA (18:1); FA (16:0); FA (16:1) | Lipid metabolism | Metabolism | 0.007881548 | 2.10339 |
| Citrate cycle (TCA cycle) | Malic acid; Fumaric acid | Carbohydrate metabolism | Metabolism | 0.009389942 | 2.02734 |
| Arginine biosynthesis | L-Glutamine; Fumaric acid | Amino acid metabolism | Metabolism | 0.012333309 | 1.90892 |

**Table S9| Continued**

| Pathway name | Related metabolites | Class Ⅱ | Class Ⅰ | *p* | -log(*p*) |
| --- | --- | --- | --- | --- | --- |
| D-Amino acid metabolism | L-Histidine; L-Glutamine; D-Glutamine | Metabolism of other amino acids | Metabolism | 0.012903273 | 1.8893 |
| Pathways in cancer | Malic acid; Fumaric acid | Cancer: overview | Human Diseases | 0.013393259 | 1.87311 |
| Glucagon signaling pathway | Malic acid; Fumaric acid | Endocrine system | Organismal Systems | 0.014491631 | 1.83888 |
| Alanine, aspartate and glutamate metabolism | L-Glutamine; Fumaric acid | Amino acid metabolism | Metabolism | 0.018011456 | 1.74445 |
| Linoleic acid metabolism | FA (20:4); FA (20:3) | Lipid metabolism | Metabolism | 0.018011456 | 1.74445 |
| Pyruvate metabolism | Malic acid; Fumaric acid | Carbohydrate metabolism | Metabolism | 0.023207624 | 1.63437 |
| ABC transporters | Taurine; L-Histidine; L-Glutamine | Membrane transport | Environmental Information Processing | 0.030766581 | 1.51192 |
| GnRH signaling pathway | FA (20:4) | Endocrine system | Organismal Systems | 0.044058729 | 1.35597 |
| Leishmaniasis | FA (20:4) | Infectious disease: parasitic | Human Diseases | 0.044058729 | 1.35597 |
